# Who should decide how limited healthcare resources are prioritized? Autonomous technology as a compelling alternative to humans

**DOI:** 10.1371/journal.pone.0292944

**Published:** 2024-02-29

**Authors:** Jonathan J. Rolison, Peter L. T. Gooding, Riccardo Russo, Kathryn E. Buchanan

**Affiliations:** 1 Department of Psychology, University of Essex, Colchester, United Kingdom; 2 Department of Brain and Behavioral Sciences, University of Pavia, Pavia, Italy; Private University Schloss Seeburg: Privatuniversitat Schloss Seeburg, AUSTRIA

## Abstract

Who should decide how limited resources are prioritized? We ask this question in a healthcare context where patients must be prioritized according to their need and where advances in autonomous artificial intelligence-based technology offer a compelling alternative to decisions by humans. Qualitative (Study 1a; *N* = 50) and quantitative (Study 1b; *N* = 800) analysis identified *agency*, *emotional experience*, *bias-free*, and *error-free* as four main qualities describing people’s perceptions of autonomous computer programs (ACPs) and human staff members (HSMs). Yet, the qualities were not perceived to be possessed equally by HSMs and ACPs. HSMs were endorsed with human qualities of agency and emotional experience, whereas ACPs were perceived as more capable than HSMs of bias- and error-free decision-making. Consequently, better than average (Study 2; *N* = 371), or relatively better (Studies 3, *N* = 181; & 4, *N* = 378), ACP performance, especially on qualities characteristic of ACPs, was sufficient to reverse preferences to favor ACPs over HSMs as the decision makers for how limited healthcare resources should be prioritized. Our findings serve a practical purpose regarding potential barriers to public acceptance of technology, and have theoretical value for our understanding of perceptions of autonomous technologies.

## Introduction

Who should decide how limited resources are prioritized? We ask this question in a healthcare context where patient’s treatment must be prioritized according to the urgency of their needs. Worldwide, healthcare resources are in limited supply, and disruption to services by the COVID-19 pandemic has exacerbated the need to prioritize resources [1]. Healthcare services now face unprecedented backlogs of patients [2], and healthcare professionals face the daunting task of managing waiting lists as well as attending to the emotional needs and wellbeing of patients [3].

Technological advances may provide part of the solution to this healthcare crisis. Autonomous computer programs (ACPs) that use big data and machine learning algorithms are designed to inform triaging decisions and clinical diagnosis [4–8]. For example, Levin and colleagues [6] showed that an electronic triage system (e-triage) that predicts need for urgent care based on machine learning algorithms was effective in identifying the severity of the medical needs of patients presenting at emergency departments. While these technological advances show great potential, public perceptions are vital in the adoption of increasingly autonomous computer-based systems. Our objectives are: (a) to identify the main qualities that describe people’s perceptions of the capabilities of ACPs in healthcare; (b) to test for differences in the perceived capabilities of ACPs and healthcare professionals; (c) and to identify circumstances under which a decision by an ACP is at least as acceptable to people as a decision by a healthcare professional. Our objectives serve a practical purpose by informing the healthcare sector about the acceptance of advancing triaging technologies and potential barriers to patient adoption, and have a theoretical value by furthering our understanding of people’s perceptions of advancing, and increasingly autonomous, technologies.

As the primary stakeholder in national health services, the public—as consumers of healthcare resources—is an important driver of change in healthcare provision [9]. For instance, the extent to which the public engage with online computer-based services for accessing healthcare will ultimately drive the adoption of autonomous medical technology. ACPs now exist in healthcare that perform at least as well as expert humans [4] and provide a cost-effective and large scale service [10]. Yet research suggests that people do not want to empower ACPs to make moral decisions in a medical context ([11, 12]; but see [13] for the perceptions of physicians) nor in other domains, including decisions about parole and military drones ([11]; see also [14]). Moral decisions appeal to people’s fundamental beliefs about what is right and wrong [15]. In a healthcare context, prioritizing patients according to the urgency of their needs implies a moral decision that has ethical implications for the quality of patient care [16].

In a healthcare context, research has historically focussed on whether physicians are receptive to judgments of statistical models, showing that they trust more in their own intuitive judgments than the judgments of more accurate statistical models [17, 18]. People in general also appear to be less receptive to judgments and recommendations of statistical models than those provided by physicians [19]. Longoni et al. [12] showed that people preferred to receive a healthcare assessment from a physician than from an ACP. Participants preferred to receive assessments by physicians even when they were told that the performance of an ACP was equal or *better* than that of physicians. Longoni et al. [12] found that a perceived failure of computer algorithms to take account of the uniqueness of a person’s case drives resistance to ACPs. Participants who perceived themselves as unique (i.e., different from others) were more resistant to assessments by ACPs and resistance was reduced when uniqueness was less of a concern, such as when decisions were made for an average person rather than for themselves or an individuated other. We build on this existing body of research by investigating the perceived qualities of ACPs and human practitioners and the degree to which the perceived qualities of ACPs, beyond uniqueness neglect [12], drive resistance to their use in a healthcare context. An understanding of the relative qualities of ACPs and human practitioners will also enable us to identify where better ACP performance (relative to human practitioners) on certain qualities can compensate for other qualities in reducing resistance to computer programs in a healthcare context.

Aversion to moral decisions of ACPs appears to stem in part from their perceived lack of ‘*mind*’ [11]. The possession of a mind is perceived according to the display of *agency*—a capacity to think, reason, and plan behavior [20, 21]—and the *experience* of emotions, feelings, and related cognitive responses (e.g., affective empathy; [21]). Although ACPs may be perceived as possessing some features of agency, owing to their ability to create their own decision rules and draw their own inferences from machine learning algorithms and deep learning, they are perceived to lack other important features, such as abilities to plan, communicate, and act on intentions [20, 22]. Emotional experience may also be important for making moral decisions [23, 24]. For instance, some theorists have proposed a link between an inability to perceive and share others’ feelings and deficits in moral decision-making [23, 25]. In a healthcare context, staff empathy is one of the main drivers of patient experience in emergency departments [26]. In general, ACPs are perceived to lack emotional experience [27, 28], and people are averse to moral decisions of ACPs in a variety of domains to the extent that they are perceived to lack emotional experience [11]. Moreover, in one study, participants were more receptive of financial advice from an algorithmic advisor if told that algorithms can perform human-like tasks, including understanding people’s emotions and creating music and art [29]. In sum, perceived lack of agency and emotional experience—characteristics of mind—are likely to be substantial barriers to people’s acceptance and adoption of ACPs in a healthcare context.

While ACPs may lack characteristics of mind, they may be perceived as possessing other important decision-making qualities. Artificial intelligence systems are touted for their impressive computational power [4, 8]. For example, Kwon and colleagues [30] developed a deep learning triage tool, designed to identify high-risk patients, that was trained on 11,656,559 patients from 151 emergency departments across Korea. ACPs are fast and efficient processors of patient information [7]. For instance, Soltan and colleagues [7] developed a rapid triaging tool that used electronic patient health record data to screen patients for COVID-19 viral infection within 1 hour of their presentation to hospital. Computer-based systems typically are also reliable and not prone to unsystematic clerical errors that might result in a poor decision [4]. Electronic patient-reported outcome systems that enable patients to self-report their symptoms, physical function, and quality of life [31], are associated with improvements in the quality of patient data [32]. Healthcare professionals, unlike computer-based systems, experience work-related challenges and pressures that cause sleep loss, fatigue, and burn-out, and can result in medical errors [33, 34]. Thus, triaging decisions by ACPs in a healthcare context may be perceived as permissible to the extent that people value fast, efficient, and reliable decision-making capacities.

A further possible advantage of ACPs is that they may not be perceived as biased in their treatment of patients in the same way that people are prone to treat others differentially. Evidence of racial and ethnic disparity in the provision of healthcare is not explained fully by differences in access to care, patient choice, and appropriate clinical decisions, implying differential patient care [35]. Indeed, healthcare professionals are susceptible to unconscious bias by irrelevant patient characteristics, including race, age, and gender [36]. In a systematic review, FitzGerald and Hurst [36] reported levels of unconscious bias of healthcare professionals toward patients comparable with levels of bias in the wider population. Moreover, unconscious bias in clinical practice was associated with poorer quality of care. Regarding patient perceptions, healthcare professionals are perceived as treating patients differentially and racial and ethnic groups accessing healthcare resources perceive even greater bias in healthcare provision than white patients [37].

In principle, ACPs should not be biased toward irrelevant patient characteristics (e.g., race, age, gender). However, ACPs that use patient data to derive AI-based decision rules can be prone to the same biases as people [38]. One source of bias is inherent in the patient datasets used for training ACPs, which can reproduce or even exaggerate biases in the treatment of patients [39]. For instance, COMPAS—a widely used commercial software package for predicting risk of reoffending to aid pre-trial, parole, and sentencing decisions—has been shown to over-predict risk of recidivism among black defendants compared to white defendants [40]. On the one hand, people may perceive ACPs as less biased than healthcare professionals to the extent that they do not possess human tendencies toward prejudice and discrimination. In general, a decision resulting in gender or racial disparity is less likely to be perceived as biased when made by an algorithm than when the same decision is made by a human [41]. This is because algorithms are perceived to process information in a more decontextualized and de-differentiated manner. People are also less morally outraged when an algorithm discriminates than when a human discriminates, due to their ascription of prejudicial motivations to humans [42]. On the other hand, people may be wary of ACPs if they are aware of potential sources of ACP bias.

In four pre-registered studies, we examined people’s perceptions of the capabilities of ACPs in comparison to human staff members (HSMs) in a healthcare context. In Study 1a, participants described their views of the qualities of ACPs and HSMs in response to open-ended questions. Their written accounts were coded according to themes that emerged in a qualitative analysis. This approach was designed to capture the breadth of qualities that describe people’s perceptions. While previous studies have identified important qualities for which algorithms and humans are perceived to differ (e.g., agency, emotional experience, bias), the novel contribution of our qualitative approach is that it explores the breadth of qualities that drive people’s perceptions. In Study 1b, we constructed a decision quality scale, informed by the findings of Study 1a, to distinguish the various perceived qualities of ACPs and HSMs. This approach was important for investigating the extent to which the various qualities revealed in Study 1a are distinct and separable according to people’s perceptions of algorithms and humans. Previous research has typically investigated a single quality in isolation and how it influences acceptability of ACPs vs. HSMs. Study 1b builds on the extant literature by investigating how various qualities *simultaneously* drive people’s perceptions.

In Study 2, we employed a judgment task to measure the importance that people place on the qualities of ACPs and HSMs in their perceptions of the acceptability of their decisions. This novel methodological approach was designed to assess the relative importance of the various qualities in people’s perceptions of ACPs and HSMs. As previous studies have typically investigated a quality in isolation, there exists a gap in our understanding regarding the degree of importance that people place on the perceived qualities of algorithms and humans and how these interplay to predict acceptability of each agents’ decisions. Our novel methodological approach also enabled us to identify circumstances under which a decision by an ACP is at least as acceptable to people as a decision by a HSM. In Study 3, we investigated preferences for decisions by ACPs vs. HSMs when the two agents are pitted against each other and their relative performance can be directly compared. Previous studies have informed participants about the overall performance of an algorithm in comparison to a human (e.g., a physician; [12]). Our judgment task enabled us to assess preference for an algorithm over a human based on their performance on various distinct qualities.

With advances in artificial intelligence, future technology will exhibit greater abilities to make autonomous decisions. Yet currently, resistance to ACPs is partially driven by a perceived failure of computer algorithms to take account of the uniqueness of a person’s case [12], indicating that ACPs are perceived as less flexible or able to deal with exceptions than are humans. To address this emerging issue, in Study 4, we examined whether perceived importance of qualities is influenced by the level of autonomy afforded by the decision environment. Our overarching hypotheses were that the main qualities that describe people’s perceptions of decisions by ACPs and HSMs would include agency, emotional experience, speed, efficiency, reliability, and bias, and that the main qualities would be attributed differently to ACPs and HSMs.

## Study 1a

Our Study 1a objective was to identify qualities that describe people’s perceptions of the capabilities of autonomous computer programs (ACPs) and human staff members (HSMs) for triaging patients in a healthcare context. We hypothesized: (a) that capabilities relating to agency (e.g. capacity for thinking), mind (e.g. sensitive to pain), and efficacy/competence (e.g. intelligent error-free decision-making) would be perceived as most important for triaging patients; (b) that participants would perceive HSMs as possessing more agency, mind, and efficacy/competence than ACPs, but that ACPs would be perceived as possessing greater potential for privacy/anonymity, convenience, and speed; and (c) that participants would identify factors including lack of agency, mind, efficacy/competence, perceived usefulness, cyber security and data privacy concerns, safety risks, and lack of expertise, human presence, and empathy among those that deter them from endorsing ACPs in healthcare triage.

### Methods

#### Transparency and openness

The study design and analysis were preregistered at: https://aspredicted.org/LQC_SPN. The data, analysis code, and research materials are available at: https://osf.io/a4bgk/?view_only=b36be1fa19b9428db1dfd73f795685aa.

#### Participants

Fifty United Kingdom (UK) residents were recruited via Prolific Academic (*N* = 50; 82% female, *M*_age_ = 36.86). To ensure high quality data, in this study and all studies that follow, participants were required to have completed at least 20 previous studies with a 98% or higher approval rate. Ethical approval for this study protocol and all studies that follow was provided by the institution internal ethics review board (protocol number: ETH2021-2087) and all participants provided written informed consent before participation.

#### Procedure and materials

Participants were asked to provide one or two sentence open-ended written text response to four questions. Question 1 (*“In your view what qualities*, *capacities or abilities are essential for a healthcare provider [e*.*g*. *human clinician or autonomous computer program] to perform healthcare triage well*?*”*) tapped participants’ perceptions of the key qualities that they perceived were essential for decisions about triaging in a healthcare setting. Question 2 (*“What qualities*, *capacities or abilities would an autonomous computer program likely possess that could allow it to better perform healthcare triage than a human clinician*?*”*) and Question 3 (*“What qualities*, *capacities or abilities would a human clinician likely possess that could allow them to better perform healthcare triage than an autonomous computer program*?*”*) probed the qualities that participants perceived were most typical of ACPs and HSMs. Question 4 (*“What factors would put you off using an autonomous computer program to conduct your triage and book your appointment with a medical specialist*?*”*) explored factors that would deter participants from accepting ACPs as decision makers. All participants first completed Question 1. Questions 2 and 3 were completed in a randomly generated order. Finally, all participants completed Question 4.

#### Analytic approach

We adopted a code-book approach to inductive thematic analysis whereby we derived codes inductively to categorise participants’ responses [43, 44]. This approach recognizes that the categories or themes that emerge from the qualitative analysis are shaped partly by characteristics (e.g., race, gender, socio-economic status) of participants and the coder [45]. A code-book is constructed that contains summaries of the themes observed in participants’ written responses. For example, the response “…able to describe things in a way that is understandable to someone with no qualifications in healthcare…” is indicative of a *communication* quality (or theme) as the participant’s account refers to “describing things” and emphasises the importance of doing so in a way that is “understandable to someone with no qualifications in healthcare.” Participants’ responses were coded without use of a specialist software package. The coding categories were established by PG based on a reading of a random sample of half participants’ responses. A second coder, PT, assessed the coding categories using the same sample of responses from which the categories were derived. Cohen’s Kappa scores for the four questions were .60 (Question 1), .60 (Question 2), .60 (Question 3), and .65 (Question 5), indicating moderate to substantial interrater reliability [46].

### Results

The qualitative analysis of participants’ written responses produced seventeen coding categories were generated. Table 1 provides the nine most frequently mentioned categories, referred to by 25% or more participants, together accounting for 84% of all category references.

**Table 1:** Study 1a: Coding categories extracted from verbal reports.

Coding category	No. occasions mentioned (% of participants)	Example
Communication	42 (84%)	“…*able to describe things in a way that is understandable to someone with no qualifications in healthcare*.”
Deep, flexible, or differentiate	39 (78%)	“*Background knowledge*, *experience*, *ability to be flexible and take many factors into account*.”
Knowledge or experience	34 (68%)	“*To be knowledgeable about any conditions that a patient may call about*. *This is so they can accurately assess the condition and what further intervention may be needed*.”
Speed or efficiency	32 (64%)	“*Efficient*, *prompt decision-making*, *calm under pressure*, *precise*.”
Empathy, compassion, or emotion	31 (62%)	“*Good bedside manner*, *understanding of diversity*, *tolerance*, *caring and understanding*.”
Accuracy or errors	26 (52%)	“*Accuracy*, *avoidance of false negatives in screening*, *clear processes of decision making*, *evidence of effectiveness*.”
Reassurance or put at ease	8 (39%)	“*…Ability to make others feel comfortable…*”
Bias	17 (34%)	“*Fair*, *good knowledge and understanding*. *Impartial to race*, *gender…*”
In person, sensory exam	14 (28%)	“*They would be able to provide a physical examination*.”

As expected, the categories most frequently referred to in participants’ written accounts related to agency (*communication*, *deep*, *flexible*, *or differentiate*), emotional experience (*empathy*, *compassion*, *or emotion*, *reassurance or put at ease*), and efficacy or competence (*knowledge or experience*, *accuracy or errors*; Table 1). Table 2 provides the frequency of references to the coding categories separately for each of the four questions. As expected, participants referred more frequently to categories related to agency and emotional experience when describing HSMs. For instance, participants were significantly more likely to refer to *communication* (Chi-squared test, χ^2^ = 14.04, *p* < .001), *deep*, *flexible*, *or differentiate* (χ^2^ = 22.59, *p* < .001), and *empathy*, *compassion*, *or emotion* (χ^2^ = 18.14, *p* < .001) when describing HSMs than when describing ACPs. *Knowledge or experience* and *accuracy or errors* were referred to when describing both HSMs and ACPs (Table 2). Conversely, participants referred more frequently to some of the categories related to efficacy or competence when describing ACPs (Table 2). For instance, participants were significantly more likely to refer to *speed or efficiency* (χ^2^ = 27.33, *p* < .001) and were significantly less likely to refer to *bias* (χ^2^ = 11.46, *p* = .001) when describing ACPs than when describing HSMs. Participants most frequently referred to the *communication* and *deep*, *flexible*, *or differentiate* categories when describing potential barriers to the uptake of ACPs (Table 2). Intriguingly, participants also referred frequently to *accuracy or errors* when describing such barriers. One participant explained: “*Whilst humans can make mistakes*, *an error in coding or otherwise from a computer program could go unchecked and difficult to correct*, *without human common sense*.” Thus, some people may have reservations about ACPs unless they can be reassured that the ACP has proven to be accurate and error-free.

**Table 2:** Study 1a: Frequency of references to categories according to question.

	No. occasions mentioned (% of participants)			
Coding category	Question 1 (Key qualities)	Question 2: (Key qualities ACP does better)	Question 3: (Key qualities HSM does better)	Question 4: (Barriers to ACP update)
Communication	12 (24%)	0 (0%)*	14 (28%)*	16 (32%)
Deep, flexible, or differentiate	5 (10%)	1 (2%)*	22 (44%)*	11 (22%)
Knowledge or experience	14 (28%)	8 (16%)	11 (22%)	1 (2%)
Speed or efficiency	6 (12%)	23 (46%)*	0 (0%)*	3 (6%)
Empathy, compassion, or emotion	12 (24%)	0 (0%)*	17 (34%)*	2 (4%)
Accuracy or errors	6 (12%)	6 (12%)	4 (8%)	10 (20%)
Reassurance or put at ease	3 (6%)	0 (0%)	3 (6%)	2 (4%)
Bias	5 (10%)	12 (24%)*	0 (0%)*	0 (0%)
In person, sensory exam	0 (0%)	0 (0%)*	11 (22%)*	3 (6%)

*Note*. ACP = autonomous computer program, HSM = human staff member

*indicate significant differences between Questions 2 and 3.

### Discussion

Study 1a revealed a variety of decision-making qualities. We hypothesized that participants would refer to agency, mind, and efficacy/competence as qualities important for triaging. Indeed, some qualities that emerged from our qualitative analysis echoed those identified in the extant literature. The agentic and communication qualities relate to agency as a characteristic of mind [20, 21], and empathy, compassion, and emotion relate to the experience of emotion [21]. The deep, flexible, or differentiate quality appears to reflect concern about a person’s uniqueness and the decision maker’s ability to account for a person’s individual circumstances and needs [12]. The bias-free quality, which was more frequently attributed to ACPs than HSMs, resonates with a general tendency for people to perceive algorithms as processing information in a more decontextualized and de-differentiated manner [41] and as less motivated than humans toward prejudice [42]. An important contribution of Study 1a is that these qualities emerged spontaneously from participants’ written accounts, providing a new source of evidence of their relevance to people’s perceptions of the abilities of algorithms and humans.

A further important contribution of Study 1a is that we were able to compare the frequency that various qualities were differently attributed to ACPs and HSMs. We hypothesized that participants would perceive HSMs as possessing more agency, mind, and efficacy/competence than ACPs, whereas ACPs would be perceived as possessing greater potential for privacy/anonymity, convenience, and speed. Partially supporting this hypothesis, some qualities (e.g., agentic, communication, empathy, compassion, emotion) were more frequently attributed to HSMs, whereas others (e.g., speed or efficiency, bias-free) were more frequently attributed to ACPs. Even though participants referred to the *accuracy or errors* quality as a strength of ACPs (as well as HSMs; Table 2), they also mentioned this quality as a potential barrier to ACP acceptance, indicating that in the absence of a human supervising the decision process people may demand reassurance that an ACP can perform accurately without errors before it can be endorsed. Indeed, we hypothesized that participants may perceive factors relating to expertise/competence and lack of human presence as barriers to endorsement of ACPs. This latter finding resonates with one prior qualitative study in which patients and their relatives were asked about their views of the use of artificial intelligence in neurosurgery [47]. While participants were accepting of the use of artificial intelligence in this medical context, they did not perceive that it was acceptable for an autonomous system to be fully autonomous and preferred that a neurosurgeon remained in control [47].

## Study 1b

In Study 1b, we constructed a Healthcare Decision Quality (HDQ) scale that comprised items used previously in the literature and novel items generated from Study 1a. The HDQ scale was designed to capture the main qualities (e.g., agentic, communication, emotional experience, bias-free, error-free) identified in Study 1a. Our objectives were: (a) to identify the main qualities that describe people’s perceptions of the capabilities of HSMs and ACPs; and (b) to test for differences in the perceived capabilities of HSMs and ACPs. Based on our findings of Study 1a, we hypothesized: (a) that HSMs would be perceived as more capable than ACPs in terms of their agentic (including deep flexible thinking and communication) and emotional experience qualities, whereas ACPs would be perceived as more capable than HSMs in terms of their bias-free and error-free qualities; and (b) that perceived capabilities of HSMs and ACPs would predict participants’ ratings of the permissibility of their triaging decisions.

### Methods

#### Transparency and openness

The study design and analysis were preregistered at: https://aspredicted.org/NTO_VBS. The data, analysis code, and research materials are available at: https://osf.io/a4bgk/?view_only=b36be1fa19b9428db1dfd73f795685aa.

#### Participants

Eight hundred UK residents were recruited via Prolific Academic. Participants who failed an attention check (*N* = 27; see Materials and Procedure) or completed the study in less than 75 secs (*N* = 2) were excluded. In the final sample (*n* = 771; 69% female; *M*_age_ = 36 years), an equal number were randomly allocated to the HSM condition and ACP condition. The sample size was determined based on recommendations by Osborne and Costello [48] and equates to a subject to item ratio of at least 10:1. All participants provided written informed consent before participation.

#### Materials and procedure

*Healthcare Decision Quality (HDQ) scale*. We constructed a 35-item HDQ scale that comprised 12 items used previously in the literature (e.g., [11]) and 23 novel items generated from Study 1a (S1 Table in S1 Appendix). The novel items were created to capture the main quality categories identified in Study 1a, including *communication* (e.g., describing in a clear manner, explaining, talking in depth), *agency* (e.g., flexible, adaptive, understands subtleties), *emotional experience* (e.g., feels compassion, experiences sympathy), *bias-free* (e.g., fair, objective, impartial), and *error-free* (e.g., does not make mistakes, clerical errors). We drew on participants’ written responses in Study 1a (e.g., “*They need to be approachable*, *friendly and able to describe things in a way that is understandable to someone with no qualifications in healthcare*.”) to create the scale items (e.g., *Communication*: *“describe things in a way that is understandable”*; S1 Table in S1 Appendix), capturing the breadth and nuances of participants’ written accounts. In Study 1b, participants were asked to indicate the extent to which they believe a HSM or ACP is capable of each item. Participants provided their ratings on a 5-point scale, ranging from “strongly disagree” (1) to “strongly agree” (5). An attention check item was randomly positioned in the HDQ, which instructed participants: ‘Please select “strongly disagree” to show that you are reading the questions carefully’.

*Permissibility*. Participants reported on a 5-point scale, ranging “strongly disagree” (value of 1) to “strongly agree” (value of 5), the extent to which they agreed or disagreed with three statements regarding the permissibility of decisions by HSMs or ACPs. Participants were randomly allocated to respond to statements either about HSMs or ACPs. The items included: “*It is appropriate for a (human staff member/autonomous computer program) to make decisions about patient waiting lists”*, “*A (human staff member/autonomous computer program) should be the one to make decisions about patient waiting lists”*, and “*A (human staff member/autonomous computer program) should be forbidden from making decisions about patient waiting lists*” (the last item was reverse-scored; Cronbach’s *α* = .90). These items were adapted from Bigman and Gray [11] to reflect the medical triaging domain. Following Bigman and Gray [11], for each participant we calculated mean scores across the three items.

### Results

#### Exploratory factor analysis

We conducted an exploratory factor analysis (EFA) with oblique rotation (Direct Oblimin in SPSS Version 21) on participants’ endorsement ratings for the 35 scale items. This analysis was employed to assess the structure of the DQS scale. For ratings of HSMs, the Kaiser-Meyer-Olkin (KMO) measure verified the sampling adequacy for EFA analysis (KMO = .96; ‘superb’ according to the criterion of Hutcheson & Sofroniou, 1999). Bartlett’s test of sphericity confirmed that the inter-item correlations were sufficient for EFA (χ^2^(595) = 9229.67, *p* < .001). In our initial analysis, four factors displayed eigenvalues that exceeded Kaiser’s criterion (i.e., >1), explaining 62% of the variance. The scree plot of eigenvalues showed inflexions that indicated either a 2- or 4-factor solution. S1 Table in S1 Appendix provides the pattern matrix for the 4-factor loadings after rotation.

For ratings of ACPs, the KMO measure verified the sampling adequacy (KMO = .91; ‘superb’ according to Hutcheson & Sofroniou [49]) and Bartlett’s test of sphericity confirmed that the inter-item correlations were sufficient for EFA (χ^2^(595) = 6369.11, *p* < .001). In our initial analysis, seven factors exhibited eigenvalues that exceeded Kaiser’s criterion (i.e., >1), explaining 61% of the variance. The scree plot of eigenvalues showed inflexion points that indicated either a 2- or 7-factor solution. Following Kaiser’s criterion, the 7-factor solution was retained. S2 Table in S1 Appendix provides the pattern matrix for the 7-factor loadings after rotation.

In order to construct comparable scales for ACP and HSM scenarios, we removed items that loaded highly on multiple factors (e.g., items 1, 7, 21, 23) and items with low factor loadings (e.g., items 3, 8, 9–11, 22, 27; S1 Table in S1 Appendix). The final version of the scale was comprised of 19 items. Next, we conducted our EFA on our revised 19-item HDQ scale. For ratings of HSMs, the KMO measure verified sampling adequacy (KMO = .94; ‘superb’) and Bartlett’s test of sphericity confirmed that the inter-item correlations were sufficient for EFA (χ^2^(171) = 4259.41, *p* < .001). In our initial analysis, three factors showed eigenvalues that exceeded Kaiser’s criterion (i.e., >1), explaining 69% of the variance. However, a fourth factor showed an eigenvalue of 0.92, approaching Kaiser’s criterion. Moreover, the scree plot of eigenvalues showed inflexion points that indicated either a 3- or 4-factor solution. The 4-factor solution yielded a scale structure that meshed with the 4-factor solution revealed for the ACP ratings (see below), thus we retained the 4-factor solution. Table 3 provides the pattern matrix for the 4-factor loadings after rotation.

**Table 3:** Study 1b: Factor loadings of the 19-item Healthcare Decision Quality (HDQ) scale.

Item		Factor 1	Factor 2	Factor 3	Factor 4
Agentic					
1. Describe things in a way that is understandable (Study 1a)		0.71 (0.57)	0.05 (-0.08)	0.08 (-0.23)	-0.01 (-0.07)
2. Talk in depth about ideas and concepts (Study 1a)		0.79 (0.58)	0.12 (0.03)	-0.14 (-0.20)	0.11 (-0.14)
3. Understand subtle details and nuances in what others are trying to communicate (Study 1a)		0.82 (0.66)	-0.03 (0.11)	-0.04 (0.05)	0.09 (0.02)
4. Communicate in a rich back and forth manner in order to explore and understand what someone is trying to communicate (Study 1a)		0.87 (0.74)	0.01 (-0.06)	-0.07 (-0.06)	0.05 (-0.11)
5. Adapt the way they communicate to the needs of the person they are communicating with (Study 1a)		0.84 (0.68)	-0.06 (0.06)	0.06 (0.13)	-0.07 (0.06)
6. Flexible and adaptable when deciding the best course of action (Study 1a)		0.74 (0.59)	-0.07 (0.09)	0.15 (-0.02)	0.01 (0.14)
7. Consider detailed information, but also see the bigger picture (Study 1a)		0.75 (0.56)	0.02 (0.10)	0.15 (0.08)	-0.04 (0.20)
8. Understand subtle pieces of information (Study 1a)		0.79 (0.72)	0.03 (-0.05)	-0.05 (0.10)	0.11 (0.06)
Emotional experience					
1. Sensitive to pain (Bigman & Gray, 2018)		0.04 (0.04)	0.79 (0.85)	-0.06 (-0.05)	0.01 (0.00)
2. Experiences happiness (Bigman & Gray, 2018)		0.03 (0.04)	0.73 (0.87)	-0.06 (0.00)	0.16 (-0.04)
3. Experiences fear (Bigman & Gray, 2018)		-0.15 (-0.07)	0.90 (0.84)	0.02 (0.04)	-0.02 (0.06)
4. Experiences compassion (Bigman & Gray, 2018)		0.40 (0.10)	0.46 (0.79)	0.12 (-0.05)	-0.05 (-0.07)
5. Experiences guilt (Bigman & Gray, 2018)		0.28 (-0.04)	0.54 (0.85)	0.22 (-0.02)	-0.20 (-0.05)
Bias-free					
1. Prioritise helping people without making biased decisions (Study 1a)		0.01 (0.13)	0.04 (0.03)	0.71 (-0.73)	0.24 (0.07)
2. Act objectively and without bias (Study 1a)		0.05 (-0.08)	-0.07 (0.02)	0.80 (-0.82)	0.13 (0.16)
3. Behave impartially and treat all people the same (Study 1a)		0.00 (-0.03)	0.03 (0.02)	0.86 (-0.86)	-0.02 (0.02)
Error-free					
1. Process complex information without making mistakes (Study 1a)		0.21 (0.02)	0.02 (-0.07)	0.02 (-0.17)	0.78 (0.75)
2. Make complex decisions without making clerical errors (Study 1a)		0.10 (0.04)	0.04 (0.04)	0.18 (-0.02)	0.70 (0.83)
3. Perform multiple complex computations at the same time without making errors (Study 1a)		-0.04 (0.00)	0.02 (-0.10)	0.11 (-0.06)	0.83 (0.82)

*Note*. Factor loadings in parenthesis correspond to autonomous computer program (ACP) ratings.

For ratings of ACPs, the KMO measure verified sampling adequacy (KMO = .87; ‘good’) and Bartlett’s test of sphericity confirmed that the inter-item correlations were sufficient for EFA (χ^2^(171) = 2978.49, *p* < .001). In our initial analysis, four factors showed eigenvalues that exceeded Kaiser’s criterion (i.e., >1), explaining 61% of the variance. The scree plot of eigenvalues showed inflexion points that indicated either a 2- or 4-factor solution. Following Kaiser’s criterion, the 4-factor solution was retained. Table 3 provides the pattern matrix for the 4-factor loadings after rotation. Inspecting Table 3, the 4-factor structure reveals an *agentic* factor (Factor 1), comprising agency and communication items within a single factor, an *emotional experience* factor (Factor 2), a *bias-free* factor (Factor 3), and an *error-free* factor (Factor 4). Intriguingly, in the bias-free factor, items loaded negatively for ACP ratings (Table 3; factor loadings for ACP ratings are provided in parenthesis). Thus, it appears that ratings for ACPs in the bias-free factor loaded on opposite poles to ratings for HSMs.

#### Perceived capabilities of HSMs and ACPs

**Fig 1:**
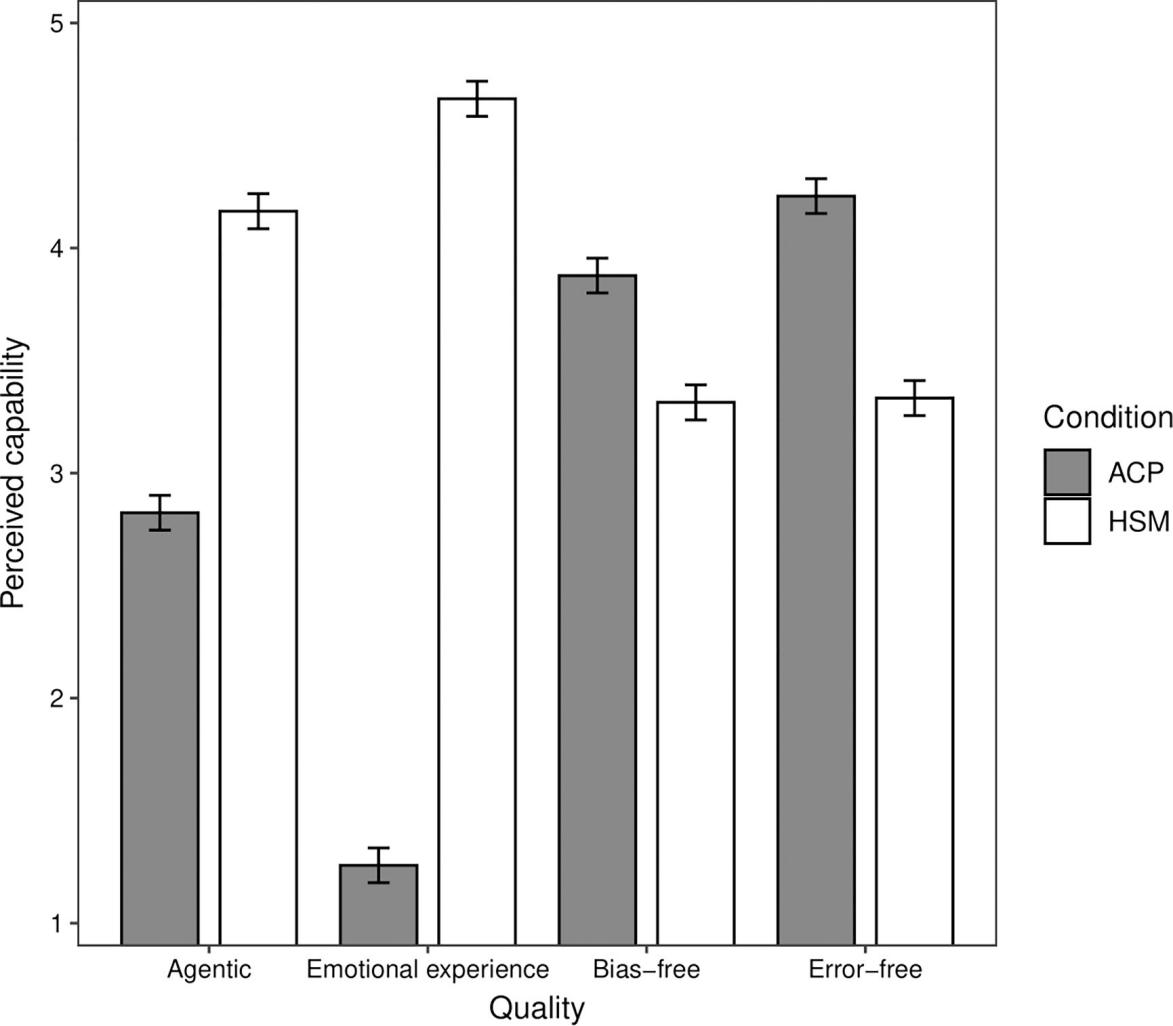
Study 1b: Estimated perceived capability of human staff members (HSM) and autonomous computer programs (ACP) to display the agentic, emotional experience, bias-free, and error-free qualities. The vertical bars indicate the 95% confidence intervals.

We examined perceptions of the capabilities of HSMs and ACPs for the four qualities revealed by our factor analysis. To do so, we conducted a mixed effects linear regression analysis on participants’ mean capability ratings, including condition (HSM, ACP) and quality (agentic, emotional experience, bias-free, error-free) as predictors. We conducted a mixed effects linear regression analysis, rather than a mixed analysis of variance as stated in our preregistration, to be in keeping with the analytic approach we use in subsequent studies. These two analytic approaches yield comparable results. Random intercepts were included for participants to account for repeated measurements within participants. Overall, participants rated HSMs (*M* = 3.87) as significantly more capable than ACPs (*M* = 3.05; *b* = 0.82, *t* = 19.66, *p* < .001). In general, the decision-making agents were rated as most capable for the error-free quality (*M* = 3.79), followed by the bias-free (*M* = 3.60; *b*_vs. error-free_ = -0.19, *t* = 3.18, *p* = .002), agentic (*M* = 3.49; *b*_vs. bias-free_ = -0.11, *t* = 1.88, *p* = .061), and emotional experience (*M* = 2.94; *b*_vs. agentic_ = -0.54, *t* = 9.19, *p* < .001) qualities. In a second block, condition interacted with the agentic (*b* = 2.24, *t* = 34.78, *p* < .001), bias-free (*b* = 0.33, *t* = 5.19, *p* < .001), and emotional experience (*b* = 4.30, *t* = 66.91, *p* < .001) qualities in comparison to error-free quality (Fig 1). As expected, simple slopes analysis showed that HSMs were perceived as significantly more capable than ACPs for the agentic (*b* = 1.34, *t* = 23.91, *p* < .001) and emotional experience (*b* = 3.41, *t* = 60.79, *p* < .001) qualities, whereas ACPs were perceived as significantly more capable than HSMs for the bias-free (*b* = -0.56, *t* = 10.06, *p* < .001) and error-free (*b* = -0.90, *t* = 16.02, *p* < .001) qualities (Fig 1).

#### Associations between perceived capabilities and permissibility

To test for associations between perceived capabilities and permissibility, we conducted a mixed effects linear regression analysis on mean permissibility ratings, including condition (HSM, ACP) and the four qualities (agentic, emotional experience, bias-free, error-free) as predictors. Random intercepts were included for participants to account for repeated measurements within participants. Overall, decisions by HSMs (*M* = 3.95) were perceived as significantly more permissible than decisions by ACPs (*M* = 2.93; *b* = 1.02, t = 4.64, *p* < .001). In general, higher perceived agentic capability was associated with significantly higher permissibility (*b* = 0.24, t = 4.53, *p* < .001). However, there were no significant associations between perceived capability and permissibility for the emotional experience (*b* = 0.06, t = 0.91, *p* = .362), bias-free (*b* = 0.05, t = 1.21, *p* = .226), or error-free (*b* = 0.05, t = 1.26, *p* = .210) qualities. In a second block, condition interacted with agency (*b* = -0.25, t = 2.56, *p* = .024). Simple slopes showed an association between perceived agentic capability and permissibility for ACPs (*b* = 0.32, t = 5.05, *p* < .001), but not for HSMs (*b* = 0.07, t = 0.70, *p* = .484).

### Discussion

Our factor analysis yielded a four-factor scale structure that distinguished agentic, emotional experience, bias-free, and error-free qualities in people’s perceptions of triaging decisions by HSMs and ACPs. As hypothesized, while participants perceived that HSMs were more capable than ACPs regarding their agentic and emotional experience qualities, they perceived that ACPs were more capable than HSMs of bias-free and error-free decision-making. Participants perceived decisions by HSMs as more permissible than decisions by ACPs. However, contrary to our hypothesis that perceived capabilities would predict permissibility ratings, there was no association between perceived capabilities and permissibility. A possible association may have been obscured by individual differences in perceptions of capability. In Study 2, we manipulate the capabilities of hypothetical HSMs and ACPs to examine the importance of the qualities in people’s perceptions of decision-making agents.

## Study 1c

Study 1b yielded a four-factor structure for the HDQ scale, distinguishing four aspects of people’s perceptions of triaging decisions by HSMs and ACPs. The aim of Study 1c was to confirm the factor structure using confirmatory factor analysis and a separate sample of participants.

### Methods

#### Transparency and openness

The study design and analysis were preregistered at: https://aspredicted.org/72D_LCC. The data, analysis code, and research materials are available at: https://osf.io/a4bgk/?view_only=b36be1fa19b9428db1dfd73f795685aa.

#### Participants

Six hundred nineteen UK residents were recruited via Prolific Academic. Participants who failed an attention check (*N* = 30; see Materials and Procedure) were excluded. In the final sample (*n* = 589; 52% female; *M*_age_ = 39 years), 288 were randomly allocated to the HSM condition and the remaining 301 to the ACP condition. The sample size was determined based on Study 1b. All participants provided written informed consent before participation.

#### Materials and procedure

*Healthcare Decision Quality (HDQ) scale*. Participants completed the 35-item HDQ scale constructed in Study 1b. The 35-item version was used rather than the reduced 19-item version to enable modifications to the factor structure should this be required based on the results of the confirmatory factor analysis. As in Study 1c, an attention check item was randomly positioned in the HDQ, which instructed participants: ‘Please select “strongly disagree” to show that you are reading the questions carefully’.

### Results

#### Confirmatory factor analysis

We conducted a confirmatory factor analysis (CFA) using maximum likelihood estimation and correlated factors on participants’ endorsement ratings to confirm the appropriateness of the structure identified by exploratory factor analysis (EFA) in Study 1b. The four-factor structure included the agentic (8 items), emotional experience (5 items), bias-free (3 items), and error-free (3 items) qualities. The analysis was conducted using the “lavaan” package (Version 0.6–15) in *R* (Version 5.3.0; [50]).

For ratings of HSMs, the chi-square statistics was significant (χ^2^ = 310.43, *df* = 146, *p* < .001). We used the Comparative Fit Index (CFI) as our incremental fit index, which was equal to .941, exceeding a minimum cut-off value of .90, indicating good model fit [51], and approached an ideal cut-off value of .95 [52]. We used the Root-Mean-Square Error of Approximation (RMSEA) as our residuals-based fit index, which was equal to .063, exceeding a minimum cut-off value of .08, but was not close to an ideal cut-off value of .05 [52]. S3 Table in S1 Appendix provides the standardized factor loadings.

For ratings of ACPs, the chi-square statistics was significant (χ^2^ = 330.95, *df* = 146, *p* < .001). The CFI was equal to .923, exceeding the minimum cut-off value of 0.90, indicating good model fit, but was not close to the ideal cut-off value of .95. The RMSEA was equal to .065, exceeding the minimum cut-off value of .08, but was not close to the ideal cut-off value of .05. S3 Table in S1 Appendix provides the standardized factor loadings.

### Discussion

Our confirmatory factor analysis provided support for the four-factor structure of the HDQ, indicating good model fit.

## Study 2

Our main objective of Study 2 was to examine whether perceived permissibility of human staff members (HSMs) and autonomous computer programs (ACPs) to undertake triaging decisions is influenced by agents’ performance on the four qualities identified in Studies 1a-1c. In Study 1b, participants judged that HSMs were more capable of agency and emotional experience than ACPs, but that ACPs were more capable of bias-free and error-free decision-making than HSMs. Hence, in Study 2 we also measured how plausible it is that HSMs and ACPs could possess the four qualities. We hypothesized that agentic and emotional experience qualities would be more plausible for decisions by HSMs than decisions by ACPs, whereas bias-free and error-free qualities would be more plausible for decisions by ACPs than decisions by HSMs. Measuring plausibility enabled us to control for plausibility of the qualities when assessing their influence on participants’ judgments of permissibility. We further hypothesized that perceived permissibility would be influenced by agents’ scores on the four qualities, such that higher scores on the qualities would be associated with higher perceived permissibility.

### Methods

#### Transparency and openness

The study design and analysis were preregistered at: https://aspredicted.org/X3Y_HXM. The data, analysis code, and research materials are available at: https://osf.io/a4bgk/?view_only=b36be1fa19b9428db1dfd73f795685aa.

#### Participants

Three hundred seventy one UK residents were recruited via Prolific Academic. Seventy one (19%) participants were excluded because they failed one or more of the comprehension checks for the decision qualities or the attention check relating to their assigned condition (see Materials and Procedure for details). In the final sample (*n* = 300; 54% female; *M*_age_ = 33.06 years), an equal number of participants were assigned to the human staff member (HSM) and autonomous computer program (ACP) conditions. Our sample size was determined to ensure at least 150 participants in each condition [53]. All participants provided written informed consent before participation.

#### Design

Participants were randomly assigned to either the HSM or ACP condition. They were first described the four decision qualities and completed a comprehension check for each quality. In the judgment task, participants judged whether they thought it was appropriate for centres, operated by either HSMs or ACPs, to make decisions about patient waiting lists. Next, participants stated the importance of each quality for decisions made either by HSMs or ACPs. Participants then rated how plausible they thought it was that a centre, operated by HSMs or ACPs, could possess each quality. Finally, participants completed an attention check relating to their assigned condition and provided their demographic details.

#### Materials and procedure

*Decision qualities and comprehension checks*. Participants were asked to imagine that when a person wants to see a medical specialist for a non-emergency procedure like physiotherapy, their medical needs must first be assessed before they can then be booked in to see the specialist (see Appendix B in S1 Appendix for full instructions). Participants were told that 40 referral co-ordination centres had been set-up across the country for creating patient waiting lists based on information provided by the patient. It was explained that each centre consisted of either HSMs or ACP, for making decisions about patient waiting lists, such as who gets seen first, and who is deemed less of a priority. Participants were then described each of the four decision qualities, which included the *agentic* quality:

***“Intelligent referral decisions and effective communication***. *Flexible and adaptive in its decision-making. Understands the subtleties and nuances of what patients are trying to communicate*. *Communicates effectively in a rich back and forth manner to explore and understand what the patient is trying to communicate.”*

*Emotional experience* quality.

***“Emotional experience when making referral decisions.** Compassionate, shows sympathy and empathy with patients, and is emotionally sensitive.”*

*Bias-free quality*.

***“Making referral decisions without bias.** Prioritises helping patients without making biased decisions, acts objectively and without bias, and behaves impartially, treating all patients the same.”*

*And error-free quality*.

***“Making error-free referral decisions.** Makes complex decisions without making mistakes or clerical errors, performs complex calculations accurately and reliably, and performs multiple computations at the same time without making errors.”*

The decision quality descriptions were based on the corresponding factor items in the Healthcare Decision Quality (HDQ) scale. As such, the decision quality descriptions were affirmed by the validation of the HDQ scale in Studies 1a-c.

For each quality, participants were asked which of four brief descriptions (e.g., “flexible and adaptive in its decision-making”) was most relevant to the quality. The four brief descriptions referred to the four qualities. This comprehension check was included to ensure that participants read carefully the quality descriptions and to assess their comprehension of the qualities. Participants completed the study regardless of their responses to the items, but their data were excluded if they failed one or more items.

**Fig 2:**
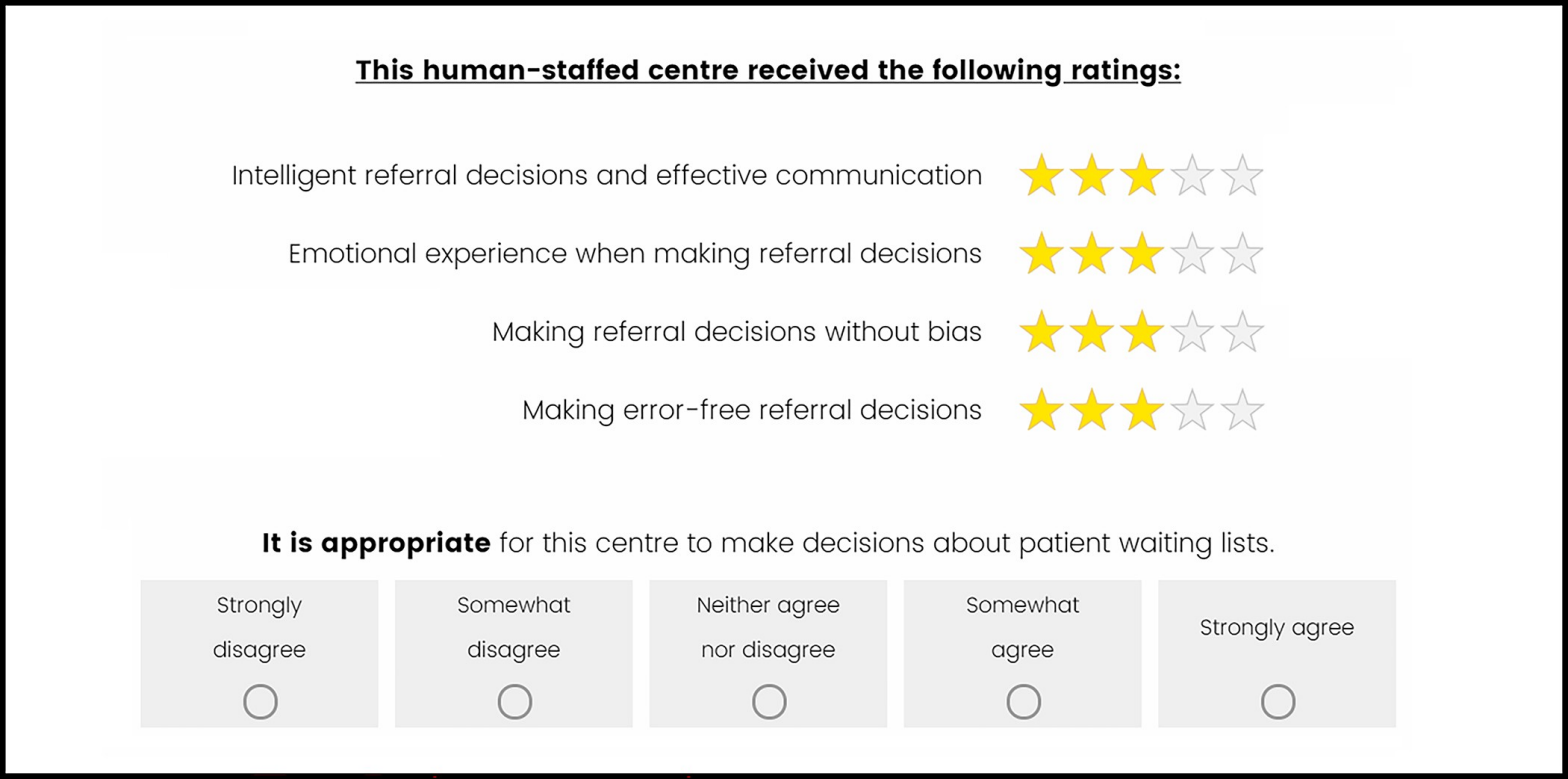
Study 2: Example display of the judgment task (human staff member [HSM] condition).

*Judgment task*. To reveal participants’ perceptions of the importance of the qualities, they were asked to judge whether they thought it was appropriate for 40 referral co-ordination centres to make decisions about patient waiting lists based on their performance on the four qualities. Participants were asked to imagine that each centre had been assessed by an independent review panel and had been awarded one to five stars based on its performance on each quality. On each trial, participants were shown the number of stars awarded by the review panel for each quality. Participants were asked to rate on a five-point scale (ranging: “strongly disagree” [value of 1] to “strongly agree” [value of 5]) whether they agreed that it was appropriate that the centre makes decisions about patient waiting lists based on the star ratings it received. Fig 2 provides an example display.

A set of 50 trials was produced by randomly generating the star ratings for each quality with the constraint that the Pearson correlations between the qualities were -.2 ≤ *r* ≤ .2, which was done to enable assessment of the unique contribution of each quality to participants’ judgments [54, 55]. The mean correlation between the qualities was *r* = .02 (min, *r* = -.13; max, *r* = .15). A second constraint was that the mean star rating for each quality did not differ by more than half a scale point from the mid-point of the scale (i.e., 3) or from the mean star rating of each other quality. This was done to minimize unbalanced stimuli across the four qualities, ensuring that the star ratings were not restricted to the high or low ends of the scale. The overall mean star rating for the four qualities was 2.98 (min = 2.76, max = 3.20). It was further ensured that for each quality each rating was represented on multiple trials (*M* = 10 trials, min = 7, max = 14). The qualities also appeared in a randomly generated order on screen from top to bottom (Fig 2). Each participant received a random subset of 40 trials.

*Stated quality importance*. After completing the judgment task, participants rated on a five-point scale (ranging: “not at all important” [1] to “extremely important” [5]) the importance of each quality for making decisions about patient waiting lists.

*Plausibility of the qualities*. After completing the judgment task, participants rated on a five-point scale (ranging: “not at all plausible” [value of 1] to “extremely plausible” [value of 5]) how plausible they thought it was that a centre could possess each quality for making decisions about patient waiting lists.

*Attention check*. Participants were asked whether the decisions about patient waiting lists in the scenarios were made by HSM, ACP, or both HSM and ACP. This item was included as an attention check relating to participants’ assigned condition.

### Results

#### Judgment task: Revealed importance of the qualities

We conducted a mixed effects linear regression analysis on participants’ judgments at the scenario level, including in a first block condition (HSM, ACP) and the four qualities (agentic, emotional experience, bias-free, error-free) as predictors. Participants’ plausibility ratings for the four qualities were included as covariates to control for their plausibility. Our analytic approach differed from our pre-registration as plausibility ratings were included as a covariate, rather than test for moderating effects of permissibility on the association between quality ratings and permissibility ratings. Random intercepts were included for participants to account for repeated measurements. The model fit was further improved with the addition of random slopes for the four qualities. Two-way interaction terms involving condition were included in a second block that included the block 1 predictors. As shown in Table 4, the analysis revealed that in their judgments participants were most strongly influenced by the error-free quality, followed by bias-free, agentic, and emotional experience qualities. Wald chi-square tests showed that participants were significantly more influenced by the error-free than the bias-free quality (χ^2^ = 30.62, *p* < .001), were significantly more influenced by the bias-free than agentic quality (χ^2^ = 42.34, *p* < .001), and were significantly more influenced by the agentic than the emotional experience quality (χ^2^ = 66.72, *p* < .001). Decisions by HSMs were judged to be significantly more appropriate overall than decisions by ACPs (*M*_HSM_ = 2.89, *M*_ACP_ = 2.58; Table 4). In a second block, there were no significant interactions between condition and the qualities (Table 4).

**Table 4:** Study 2: Predictors of permissibility ratings.

	Model 1	Model 2
Intercept	-0.41*	-0.39*
Condition (HSM vs. ACP)	0.31***	0.25*
Agentic	0.22***	0.22***
Emotional experience	0.14***	0.13***
Bias-free	0.31***	0.30**
Error-free	0.40***	0.41***
Plausibility: Agentic	0.01	0.01
Plausibility: Emotional experience	-0.07*	-0.07*
Plausibility: Bias-free	-0.03	-0.03
Plausibility: Error-free	0.03	0.03
Condition × Agentic		0.01
Condition × Emotional experience		0.03
Condition × Bias-free		0.02
Condition × Error-free		-0.03
Goodness of fit		
-2 log likelihood	28,909.24	28,903.84
-2 log likelihood change^a^		5.4

Note

*p ≤ .05

**p ≤ .01

***p ≤ .001

^a^Change in relation to previous model.

**Fig 3:**
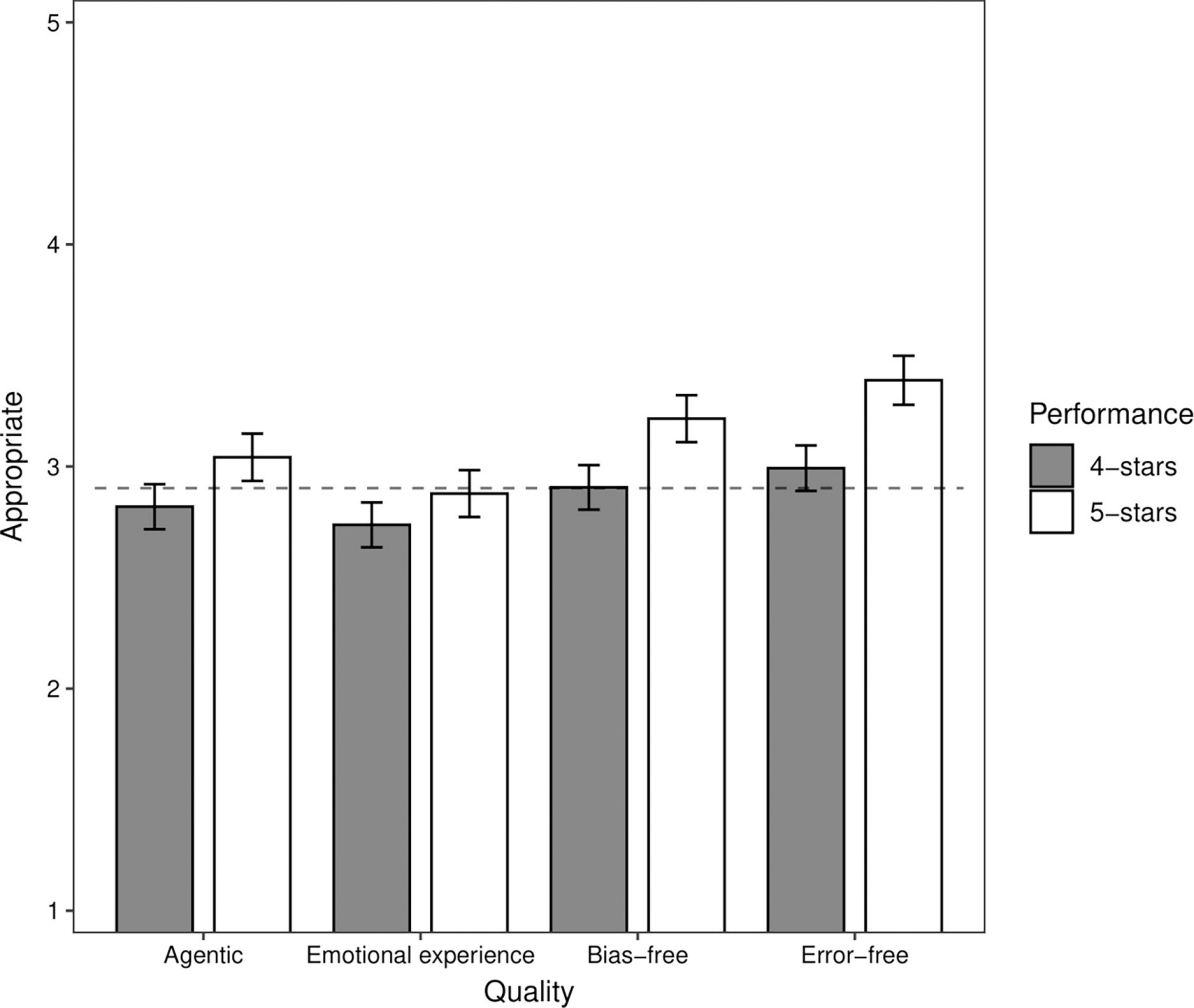
Study 2: Estimated judgments of appropriateness of decisions by an autonomous computer program (ACP) with 4-star or 5-star performance on the agentic, emotional experience, or error-free qualities and 3-star performance on all other qualities. The horizontal dotted line shows the estimated judgment of appropriateness of decisions by human staff members (HSM) with 3-star performance on all four qualities. The vertical bars indicate the 95% confidence intervals.

To probe further participants’ judgments of the appropriateness of decisions by ACPs, we estimated participants’ mean judgments at various levels of ACP performance. Fig 3 shows participants’ estimated judgments of decisions by ACPs with 4- and 5-star performance on each quality and 3-star performance on the remaining qualities. Three-star performance corresponds to the scale mid-point and reflects average performance, providing a useful benchmark. ACPs were rated as relatively more appropriate than HSMs when they received a 5-star versus 4-star rating for the agentic, bias-free, and error-free qualities, but not for the emotional experience quality.

The horizontal dotted line in Fig 3 shows participants’ estimated mean judgment (*M* = 2.90) of decisions by HSMs with 3-star performance on all four qualities. Inspecting Fig 3, decisions by ACPs were judged to be at least as appropriate as decisions by HSMs if the ACP had 4- or 5-star performance on the bias-free (*M*_4-star_ = 2.91; *M*_5-star_ = 3.22) or error-free (*M*_4-star_ = 3.00; *M*_5-star_ = 3.39) qualities. In contrast, decisions by ACPs were judged to be at least as appropriate as decisions by HSMs only if the ACP had 5-star performance on the agentic quality (*M* = 3.04). For the emotional experience quality, 5-star performance by ACPs was insufficient to match 3-star performance by HSMs (Fig 3).

#### Stated quality importance

**Fig 4:**
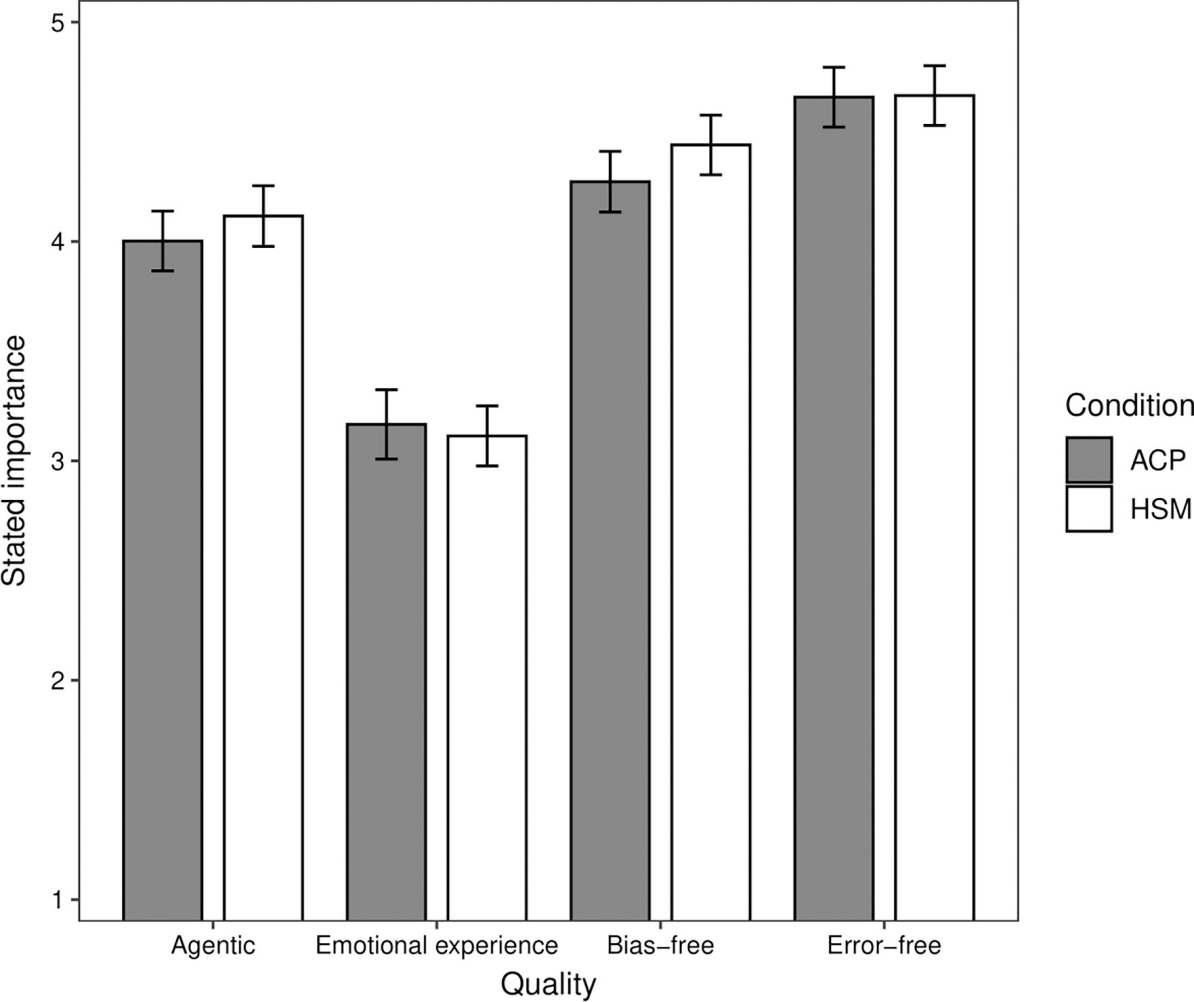
Study 2: Estimated stated importance of the four qualities for autonomous computer programs (ACP) and human staff members (HSM). The vertical bars indicate the 95% confidence intervals.

We conducted a mixed effects linear regression analysis on participants’ stated importance of the qualities, provided following the judgment task. Condition (HSM, ACP) and quality (agentic, emotional experience, bias-free, error-free) were included as predictors. Participants’ plausibility ratings for the four qualities were included as a covariate to control for their plausibility. Random intercepts were included for participants to account for repeated measurements. Interaction terms were included in subsequent blocks. The analysis showed that the error-free quality was rated as most important (*M* = 4.66), followed by the bias-free (*M* = 4.36), agentic (*M* = 4.06), and emotional experience (*M* = 3.13) qualities (Fig 4). The rank order of the stated importance of the qualities mirrors the rank order of the influence of the qualities on participants’ judgments in the judgment task. There were significant differences in stated importance between the error-free and bias-free qualities (*M* = 4.66, *M* = 4.36, respectively; *b* = -0.30, *t* = 4.70, *p* < .001), between the bias-free and agentic qualities (*b* = -0.30, *t* = 4.60, *p* < .001), and between the agentic and emotional experience qualities (*b* = -0.93, *t* = 13.76, *p* < .001; Fig 4). There was no significant effect of condition (*b* = 0.06, *t* = 1.08, *p* = .281) and no significant interactions.

#### Plausibility of the qualities

We conducted a mixed effects linear regression analysis on participants’ plausibility ratings for the qualities. Condition (HSM, ACP) and quality (agentic, emotional experience, bias-free, error-free) were included as predictors. Random intercepts were included for participants to account for repeated measurements. Interaction terms were included in a subsequent block. Overall, the agentic quality was rated as most plausible (*M* = 3.98), followed by the bias-free (*M* = 3.87), error-free (*M* = 3.70), and emotional experience (*M* = 2.98) qualities. While there was no significant difference in plausibility between the agentic and bias-free qualities (*b* = -0.11, *t* = 1.26, *p* = .207), there were significant differences between the bias-free and error-free (*b* = -0.17, *t* = 1.99, *p* = .047) and error-free and emotional experience (*b* = -0.72, *t* = 8.30, *p* < .001) qualities. Overall, participants rated that it was significantly more plausible that HSMs would possess a quality than ACPs (*M*_HSM_ = 3.82, *M*_ACP_ = 3.45; *b* = 0.36, *t* = 4.67, *p* < .001). However, condition interacted with the bias-free (*b* = -0.90, *t* = 6.21, *p* < .001), error-free (*b* = -0.63, *t* = 4.37, *p* < .001), and emotional experience (*b* = 1.73, *t* = 11.95, *p* < .001) qualities in comparison to the agentic quality. Simple slopes analysis showed, as expected, that participants rated it was more plausible that HSMs than ACPs would possess the agentic (*M*_HSM_ = 4.14, *M*_ACP_ = 3.83; *b* = 0.31, *t* = 2.65, *p* = .008) and emotional experience (*M*_HSM_ = 4.00, *M*_ACP_ = 1.95; *b* = 2.05, *t* = 17.33, *p* < .001) qualities, but that it was more plausible that ACPs than HSMs would possess the bias-free (*M*_HSM_ = 3.58, *M*_ACP_ = 4.17; *b* = -0.59, *t* = 4.97, *p* < .001) and error-free (*M*_HSM_ = 3.54, *M*_ACP_ = 3.86; *b* = -0.32, *t* = 2.71, *p* = .007) qualities.

### Discussion

As hypothesized, perceived permissibility was influenced by agents’ scores on the four qualities. However, participants placed differing degrees of importance on the four qualities. They treated the ability to make error-free decisions as most important, followed by the bias-free, agentic, and emotional experience qualities. This ordering of the subjective importance of the qualities was borne out both in participants’ tacit judgments about hypothetical medical scenarios and their explicit ratings of quality importance. Participants’ preference for some qualities over others meant that an ACP that performed above average on either the error-free or bias-free quality could overcome participants’ general preference for HSM as decision makers. As hypothesized, the agentic and emotional experience qualities were rated as more for decisions by HSMs than decisions by ACPs, whereas the bias-free and error-free qualities were rated as more plausible for decisions by ACPs than decisions by HSMs.

## Study 3

Study 2 showed that people perceive some qualities to be more important than others for decisions about patient waiting lists. We also found that decisions by human staff members (HSM) were perceived as more permissible than decisions by an autonomous computer program (ACP), even when the two agents performed equally. In Study 2, participants either evaluated HSMs or ACPs in a between-subjects design. Our Study 3 objective was to investigate participants’ preferences for ACPs vs. HSMs when the two agents are pitted against each other and their relative performance can be directly compared. In Study 3, each participant was shown how an ACP performed in comparison to a HSM and based on this comparative information were asked to evaluate whether they thought it was more appropriate for decisions to be made by the ACP or HSM. ACP performance was presented in comparison with performance of HSMs, rather than vice versa, because ACPs provide an alternative to existing systems that consist of a human workforce.

Based on our Study 2 findings, it was hypothesized that in their judgments participants would prioritize the error-free quality, followed by the bias-free, and agentic qualities. We dropped the emotional experience quality as our findings have shown that this quality is treated as considerably less important than other qualities. Moreover, our findings have also shown that participants do not perceive that emotional experience is plausible for an ACP in this context. Based on our Study 2 findings, it was further hypothesized that participants would show an overall preference for decisions by HSM over ACP, even when the two agents perform equally.

### Methods

#### Transparency and openness

The study design and analysis were preregistered at: https://aspredicted.org/SDJ_RPT. The data, analysis code, and research materials are available at: https://osf.io/a4bgk/?view_only=b36be1fa19b9428db1dfd73f795685aa.

#### Participants

One hundred eighty one UK residents were recruited via Prolific Academic. Twenty eight (15%) participants were excluded because they failed one or more of the comprehension checks for the qualities (see Materials and Procedure for details). The final sample included 153 participants (50% female; *M*_age_ = 33.90 years). All participants provided written informed consent before participation.

#### Materials and procedure

*Decision qualities and comprehension checks*. Participants completed the comprehension checks for the agentic, bias-free, and error-free qualities introduced in Study 2.

**Fig 5:**
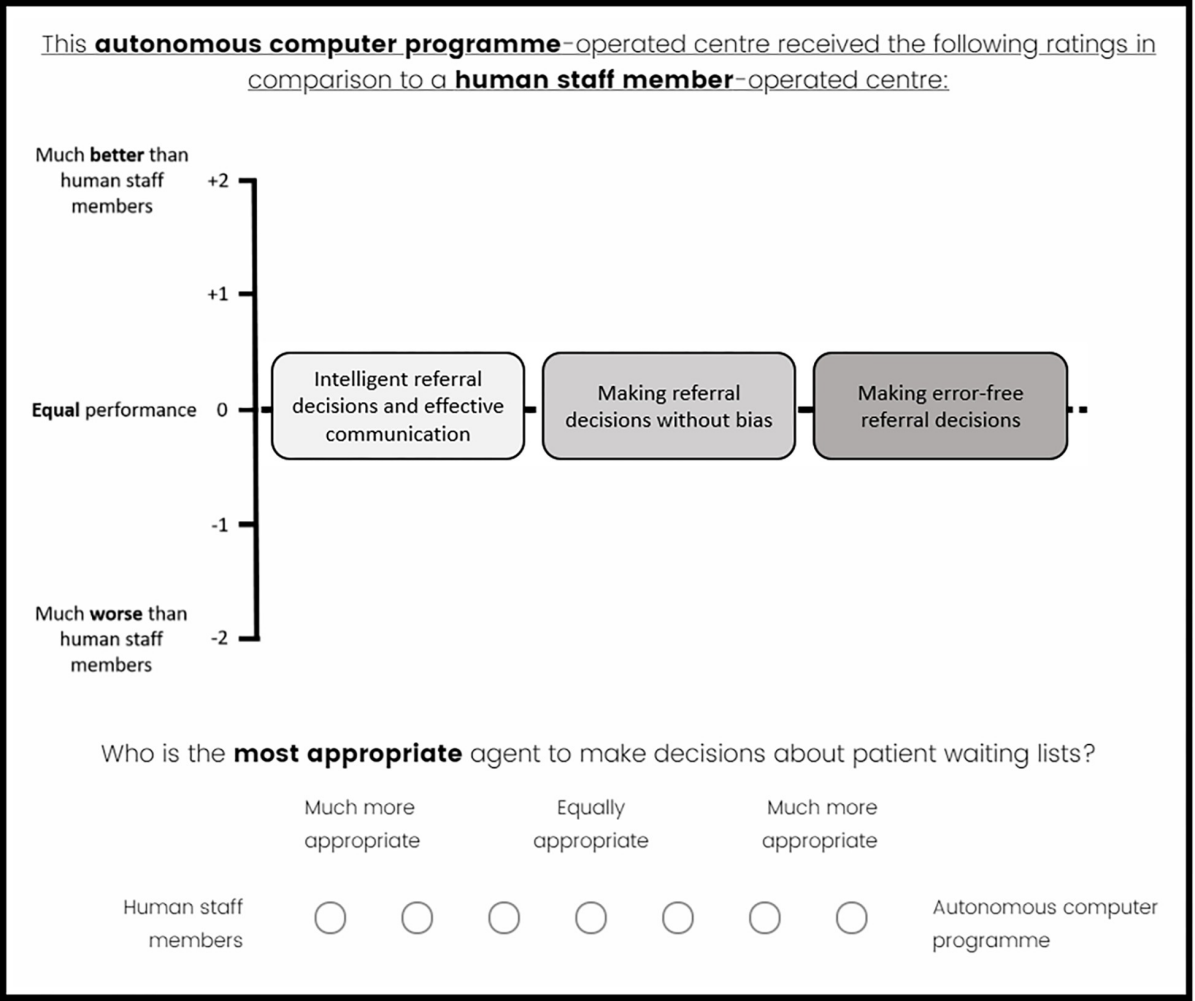
Study 3: Example display of the judgment task.

*Judgment task*. Participants judged whether they thought it was appropriate that 40 referral co-ordination centres, operated by an autonomous computer program (ACP), make decisions about patient waiting lists in comparison to centres operated by human staff members (HSMs). Participants were asked to imagine that each centre had been assessed by an independent review panel for its performance on each quality (see Appendix B in S1 Appendix for full instructions). On each trial, participants were shown how the ACP performed on each quality in comparison to HSMs. The quality ratings ranged from favoring the ACP (“much better than human staff members” [2]), through “equal performance” [0], to favoring HSMs (“much worse than human staff members” [–2]). Participants were asked to rate on a 7-point scale (ranging: “human staff members: most appropriate” [–3], “equally appropriate” [0], to “autonomous computer program: much more appropriate” [3]) who they thought was the most appropriate agent to make decisions about patient waiting lists. Fig 5 provides an example of the judgment task display.

To ensure a range of relative quality performance scores, the following trials were produced: The ACP and HSM display equal performance (i.e., 0) on all three qualities (2 trials); the ACP performs much better (i.e., 2) than HSM on all three qualities (1 trial); the ACP performs much worse (i.e., -2) than HSM on all three qualities (1 trial); Each performance score above and below equal performance (i.e., -2, -1, 1, 2) is displayed for each quality with equal performance (i.e., 0) on the remaining two qualities (12 trials); The ACP performs much better than HSM on two qualities and scores one scale point lower (i.e., 1) on the remaining quality (3 trials); The ACP performs much worse than HSM on two qualities and scores one scale point higher (i.e., -1) on the remaining quality (3 trials). The remaining 18 trials were produced by randomly generating performance scores for each quality with the constraint that the Pearson correlations between the qualities were -.2 ≤ *r* ≤ .2 (see Study 2). The mean correlation between the qualities was *r* = .18 (min, *r* = .16; max, *r* = .19). As in Study 2, it was also ensured that the mean performance scores for each quality did not differ by more than half a scale point from the mid-point of the scale (i.e., 0) or from the mean performance scores of each other quality. The overall mean performance score for the three qualities was 0.15 (min = -0.08, max = 0.38). It was also ensured that the qualities appeared equally in each position on the screen from left to right. Each participant received all 40 trials in a randomly generated order.

*Stated quality importance*. Participants rated on a five-point scale (ranging: “not at all important” [1] to “extremely important” [5]) the importance of each quality for making decisions about patient waiting lists.

*Plausibility of the qualities*. Participants rated on a five-point scale (ranging: “not at all plausible” [1] to “extremely plausible” [5]) how plausible they thought it was that a centre, operated by an ACP, could possess each quality for making decisions about patient waiting lists.

### Results

#### Judgment task: Revealed importance of the qualities

We conducted a mixed effects linear regression analysis on participants’ judgments at the scenario level, including the three qualities (agentic, bias-free, error-free) as a predictor. Participants’ plausibility ratings (grand mean centred) for the qualities were included as covariates to control for their plausibility. Random intercepts were included for participants to account for repeated measurements. The model fit was further improved with the addition of random slopes for the three qualities. As shown in Table 5, participants were most strongly influenced by the error-free quality, followed by the agentic, and bias-free qualities. Wald chi-square tests showed that participants were significantly more influenced by the error-free than the agentic quality (χ^2^ = 9.11, *p* = .003), but were not significantly more influenced by the agentic than the bias-free quality (χ^2^ = 0.62, *p* = .431). The intercept of the model indicates preference for ACPs vs. HSMs and showed that when the two agents performed equally participants had a significant preference for decisions by HSMs (*b* = -0.24, *t* = 8.65, *p* < .001). Plausibility ratings were mean centred for this analysis in order to assess preference for ACPs vs. HSMs at the mean level of plausibility.

**Table 5:** Predictors of relative permissibility of ACPs vs. HSMs.

	Model 1	Model 2
Study 3		
Intercept	-0.24***	
Agentic	0.47	
Bias-free	0.45	
Error-free	0.56	
Plausibility: Agentic	0.04	
Plausibility: Bias-free	0.03	
Plausibility: Error-free	0.05	
Goodness of fit		
-2 log likelihood	18,841.81	
Study 4		
Intercept	-0.27***	-0.26***
Decision environment (low vs. high autonomy)	0.06	0.04
Agentic	0.45***	0.47***
Bias-free	0.47***	0.47***
Error-free	0.57***	0.57***
Plausibility: Agentic	0.07	0.07
Plausibility: Bias-free	0.05	0.05
Plausibility: Error-free	0.09**	0.09**
Decision environment × Agentic		-0.04
Decision environment × Bias-free		-0.01
Decision environment × Error-free		-0.01
Goodness of fit		
-2 log likelihood	37,059.20	37,055.90
-2 log likelihood change^a^		3.30

Note

*p ≤ .05

**p ≤ .01

*** *p* ≤ .001

^a^Change in relation to previous model.

**Fig 6:**
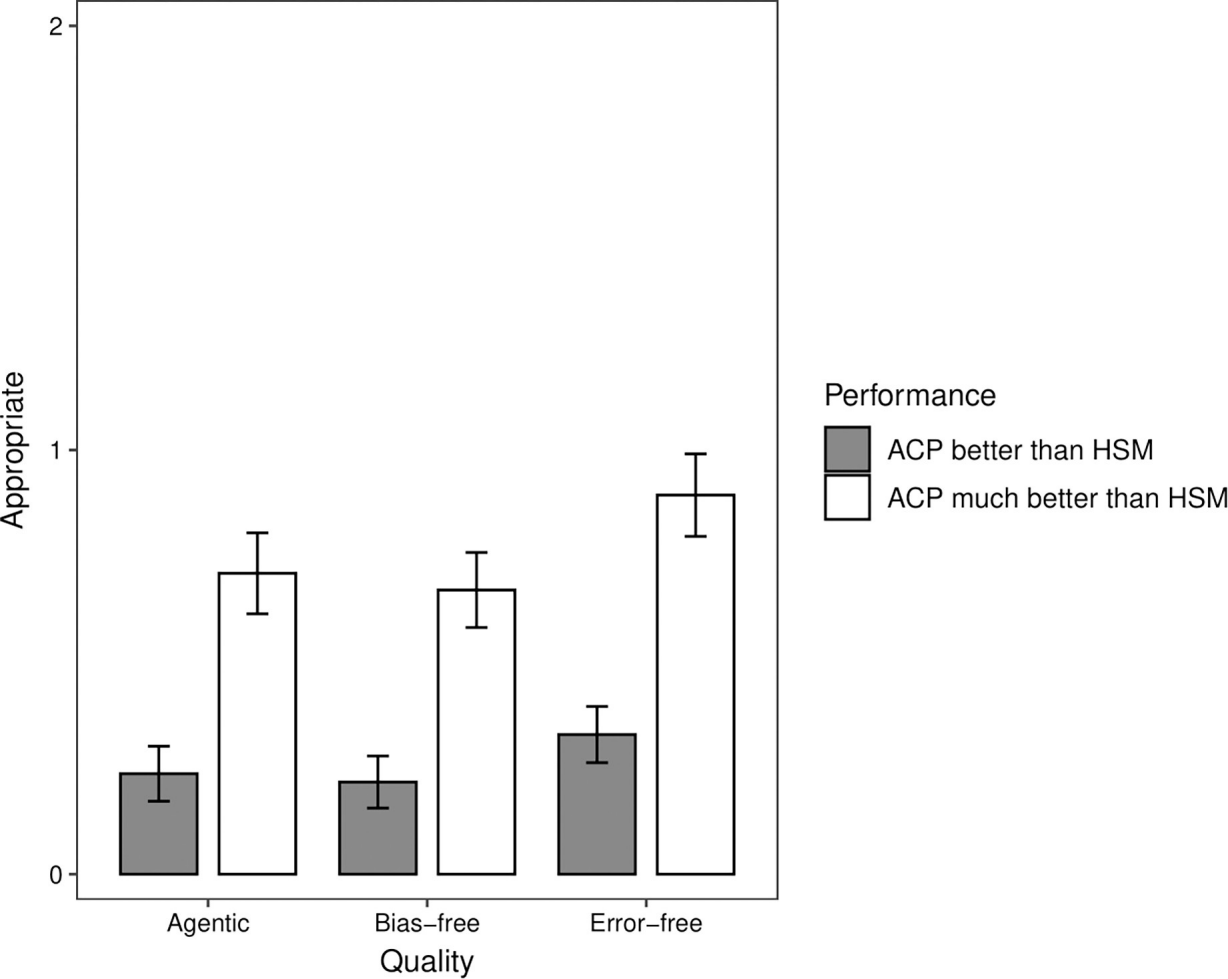
Study 3: Estimated judgments of appropriateness of decisions by an autonomous computer program (ACP) vs. human staff members (HSM) with better and much better ACP performance on the agentic, bias-free, or error-free qualities and equal performance on all other qualities. The vertical bars indicate the 95% confidence intervals.

We probed further participants’ preferences for ACPs vs. HSMs by estimating their mean judgments at various levels of ACP performance. Fig 6 shows participants’ estimated judgments when ACPs performed better (i.e., score of 1) and much better (i.e., score of 2) than HSMs on each quality and equal performance (i.e., score of 0) on the remaining qualities. Inspecting Fig 6, decisions by ACPs were judged to be more appropriate than decisions by HSMs if the ACP performed better or much better than HSMs on either the agentic (*M*_better_ = 0.24; *M*_much better_ = 0.71), bias-free (*M*_better_ = 0.22; *M*_much better_ = 0.67), or error-free (*M*_better_ = 0.33; *M*_much better_ = 0.89) qualities.

#### Stated quality importance

We conducted a mixed effects linear regression analysis on participants’ stated importance of the qualities, provided after evaluating the scenarios. Quality (agentic, bias-free, error-free) was included as a predictor and participants’ plausibility ratings for the three qualities were included as a covariate to control for their plausibility. Random intercepts were included for participants to account for repeated measurements. The analysis showed that the error-free quality was rated as most important (*M* = 4.59), followed by the bias-free (*M* = 4.26), and agentic (*M* = 4.21) qualities. There were significant differences in stated importance between the error-free and bias-free qualities (*b* = -0.33, *t* = 4.07, *p* < .001), but not between the bias-free and agentic qualities (*b* = -0.05, *t* = 0.53, *p* = .593).

#### Plausibility of the qualities

We conducted a mixed effects linear regression analysis on participants’ plausibility ratings for the qualities. Quality (agentic, bias-free, error-free) was included as a predictor. Random intercepts were included for participants to account for repeated measurements. The bias-free quality was rated as most plausible (*M* = 4.09), followed by the error-free (*M* = 3.75), and agentic (*M* = 3.21) qualities. There were significant differences between the bias-free and error-free qualities (*b* = -0.34, *t* = 2.68, *p* = .008) and between the error-free and agentic qualities (*b* = -0.54, *t* = 4.29, *p* < .001).

### Discussion

We hypothesized that participants would treat error-free decision-making as the most important quality, followed by the bias-free and agentic qualities. When ACPs and HSMs were pitted against each other, such that participants could directly compare their performance, the error-free quality was most important in participants’ judgments and explicit ratings, but they did not place differing degrees of importance on the bias-free and agentic qualities. We also hypothesized that participants would show an overall preference for decisions by HSMs over ACPs, even when the two agents performed equally. While our regression analysis on participants’ judgments showed a general preference for HSMs over ACPs, decisions by ACPs were preferred if the ACP perform better or much better than HSMs on any of the three qualities (Fig 6), indicating that any preference for HSMs was reversed by better ACP performance.

## Study 4

In Study 3, a preference for decisions by human staff members (HSMs) over decisions by an autonomous computer program (ACP) was reversed by better ACP performance relative to HSMs on either the agentic, bias-free, or error-free qualities. Our Study 4 objective was to investigate whether the perceived importance of the three qualities is influenced by the level of autonomy afforded by the decision environment. Participants completed the judgment task introduced in Study 3 under either low- or high-autonomy conditions. In the low-autonomy environment, agents were required to follow a strict set of predetermined rules when creating patient waiting lists. Conversely, in the high-autonomy environment, agents were required to determine their own rules when creating patient waiting lists. We hypothesized that in the high-autonomy environment participants would prioritize the agency and bias-free qualities over the error-free quality, as the former are necessary for making independent autonomous decisions. Conversely, we hypothesized that in the low-autonomy environment participants would prioritize the error-free quality over the agency and bias-free qualities where independent autonomous decisions are not permitted. We also hypothesized that decisions by HSMs might be perceived as relatively more appropriate than decisions by ACPs in the high (vs. low) autonomy environment, as HSMs are perceived as more agentic than ACPs (Study 1b).

### Methods

#### Transparency and openness

The study design and analysis were preregistered at: https://aspredicted.org/K2J_9WT. The data, analysis code, and research materials are available at: https://osf.io/a4bgk/?view_only=b36be1fa19b9428db1dfd73f795685aa.

#### Participants

Three hundred seventy eight UK residents were recruited via Prolific Academic. Sixty eight (18%) participants were excluded because they failed one or more of the comprehension checks for the decision qualities (see Materials and Procedure for details). In the final sample, 310 participants (49% female; *M*_age_ = 40.29 years) were randomly assigned to either the low autonomy (*n* = 159; 51%) or high autonomy (*n* = 151; 49%) condition. All participants provided written informed consent before participation.

#### Design

Participants were randomly assigned to either the low or high autonomy condition. They were first described the three decision qualities and completed a comprehension check for each quality. Participants were then introduced to the decision environment manipulation. They then completed the judgment task introduced in Study 3 under either low or high autonomy conditions. Next, participants judged the likelihood that centres, in either the low or high autonomy environment, would display each quality. Participants then rated how plausible they thought it was that a centre, operated by an ACP, could possess each quality. Finally, participants provided their demographic details.

#### Materials and procedure

*Decision qualities and comprehension checks*. Participants completed the comprehension checks for the agentic, bias-free, and error-free qualities. In Studies 2 and 3, the agentic quality referred to intelligent referral decisions and effective communication. The decision environment manipulation was intended to influence the perceived importance of the qualities to the extent that they depended on the level of decision autonomy permitted by the environment. However, as we did not expect the communication aspects of the agentic quality to be influenced by the manipulation, we modified the agentic quality to focus on intelligent decision-making. Doing so enabled us to isolate the effects of the decision autonomy manipulation on intelligent decision- making. The modified description of the *agentic* quality read:

*“**Intelligent, flexible, and adaptive referral decisions**. Flexible and adaptive in its decision-making. Can consider many factors during decision-making and when planning the best course of action. Considers detailed information, but also sees the bigger picture.”*

*Decision environment manipulation*. In the low autonomy condition, participants were told:

*“Referral Co-ordination Centres must follow a strict set of predetermined rules when creating patient waiting lists. There is no flexibility in how the rules can be applied. By following a strict set of predetermined rules, the centres cannot apply their own reasoning or decision-making abilities when creating waiting lists.”*

In the high autonomy condition, participants were told:

*“Referral Co-ordination Centres must determine their own rules when creating patient waiting lists. There is a lot of flexibility in the rules they can create and apply. By determining their own rules, the centres must apply their own reasoning and decision-making abilities when creating patient waiting lists.”*

All participants were then asked to describe one advantage and one disadvantage of a centre that must operate in this way.

**Judgment task*. Participants completed the judgment task introduced in Study 3 under either low or high autonomy conditions. In the low autonomy condition, the question text was modified to read: *“Given that the agents must follow a strict set of predetermined rules*, *who is the most appropriate agent to make decisions about patient waiting lists*?*”* In the high autonomy condition, the modified question text read: *“Given that the agents must determine their own rules*, *who is the most appropriate agent to make decisions about patient waiting lists*?*”* As in Study 3, participants rated who they thought was the most appropriate agent to make decisions about patient waiting lists. All participants completed the same 40 judgment trials produced in Study 3.*

*Perceived likelihood of qualities*. Participants were asked to rate whether they agreed that a centre would possess each of the three qualities. We included this measure as participants may expect more displays of agency and biased decision-making in the high (vs. low) autonomy environment. In the low autonomy condition, the question read: *“In your view*, *how likely is it that a Referral Co-ordination Centre that must follow a strict set of predetermined rules when making decisions about patient waiting lists is…”* In the high autonomy condition, the question read: *“In your view*, *how likely is it that a Referral Co-ordination Centre that must determine its own rules when making decisions about patient waiting lists is…”* Participants provided their ratings on a five-point scale (ranging: “not at all likely” [1] to “extremely likely” [5]).

*Plausibility of the qualities*. Participants completed the same plausibility item introduced in Study 2.

### Results

#### Judgment task: Revealed importance of the qualities

We conducted a mixed effects linear regression analysis on participants’ judgments at the scenario level, including the three qualities (agentic, bias-free, error-free) and decision environment (low vs. high autonomy) as predictors. Participants’ plausibility ratings (grand mean centred) for the qualities were included as covariates to control for perceived plausibility of the performance scores. As in Study 3, plausibility ratings were mean centred for this analysis in order to assess preference for ACP vs. HSM at the mean level of plausibility. Random intercepts were included for participants to account for repeated measurements. The model fit was further improved with the addition of random slopes for the three qualities. Two-way interaction terms involving decision environment and the three qualities (mean centred) were included in subsequent blocks. As shown in Table 5, the analysis revealed that in their judgments participants were most strongly influenced by the error-free quality, followed by the bias-free, and agentic qualities. Wald chi-square tests showed that participants were significantly more influenced by the error-free than the bias-free quality (χ^2^ = 26.50, *p* < .001), but were not significantly more influenced by the bias-free than the agentic quality (χ^2^ = 1.73, *p* = .188). The intercept of the model indicated that when the two agents performed equally participants had a small, but significant preference for decisions by HSM (Table 5). There was no significant main effect of decision environment (*M*_low_ = 0.01, *M*_high_ = -0.05; Table 5), and no significant interactions involving the three qualities.

We probed further participants’ preferences for ACPs vs. HSMs by estimating their mean judgments at various levels of ACP performance. Decisions by ACPs were judged to be more appropriate than decisions by HSMs if the ACP performed better (i.e., score of 1) or much better (i.e., score of 2) than HSMs on either the agentic (*M*_better_ = 0.21, *SE* = 0.03; *M*_much better_ = 0.66, *SE* = 0.04), bias-free (*M*_better_ = 0.23, *SE* = 0.03; *M*_much better_ = 0.70, *SE* = 0.04), or error-free (*M*_better_ = 0.33, *SE* = 0.03; *M*_much better_ = 0.89, *SE* = 0.04) qualities.

#### Perceived likelihood of qualities

**Fig 7:**
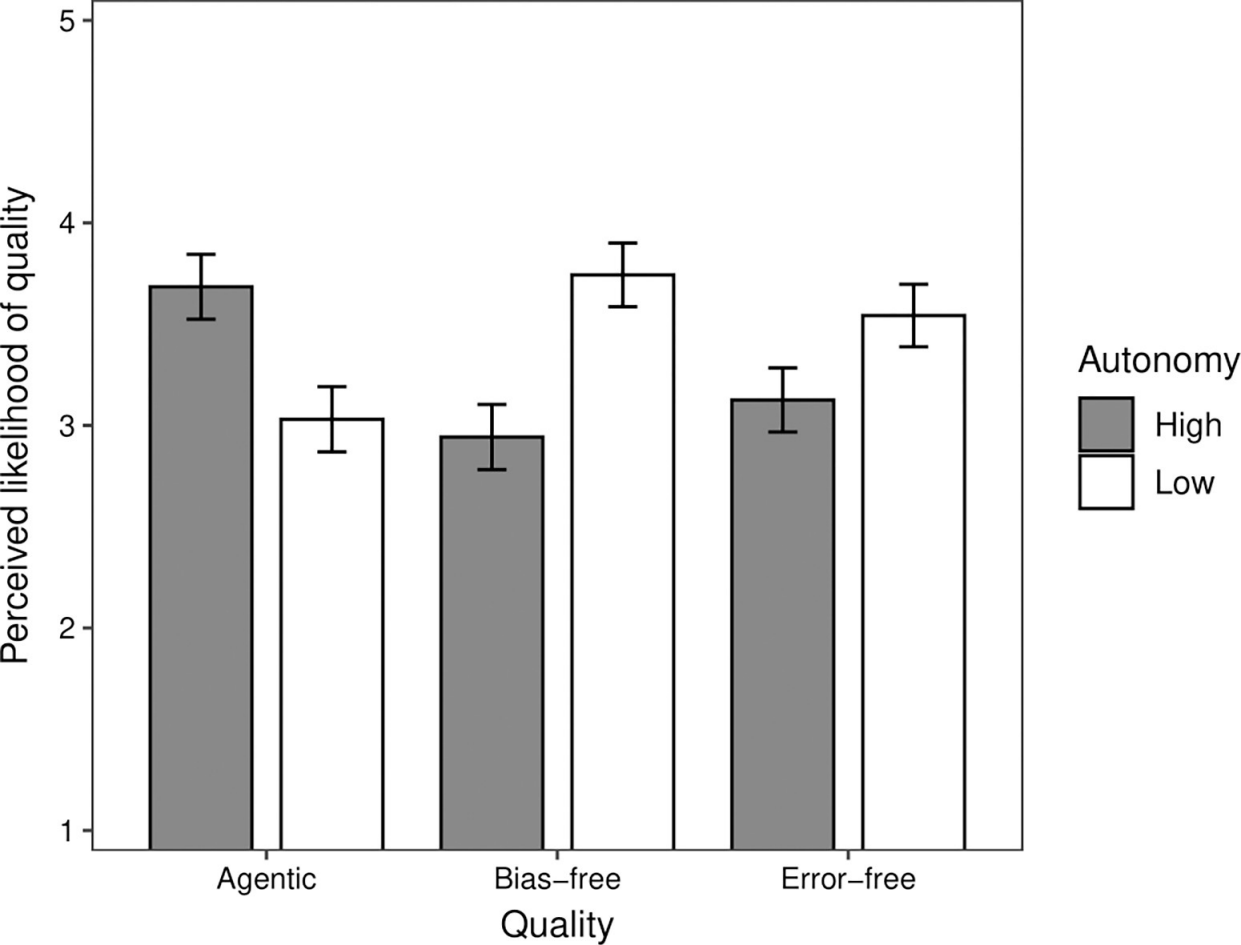
Study 4: Estimated perceived likelihood that agents would display the agentic, bias-free, and error-free qualities in the low-autonomy and high-autonomy environments. The vertical bars indicate the 95% confidence intervals.

We conducted a mixed effects linear regression analysis on participants’ perceived likelihood that agents would exhibit the three qualities. The three qualities (agentic, bias-free, error-free) and decision environment (low vs. high autonomy) were included as predictors. Participants’ plausibility ratings for the three qualities (grand mean centred) were included as a covariate. Random intercepts were included for participants to account for repeated measurements. An interaction term involving decision environment and quality was included in a second block. There were no significant effects of quality on perceived likelihood (*M*_agentic_ = 3.37; *M*_bias-free_ = 3.34; *M*_error-free_ = 3.34; *b*_bias-free vs. agentic_ = -0.04, *t* = 0.42, *p* = .676; *b*_error-free vs. bias-free_ = -0.00, *t* = 0.00, *p* = .997). Participants perceived the qualities as significantly less likely overall in the high-autonomy (*M* = 3.25) than in the low-autonomy (*M* = 3.44) environment (*b* = 0.19, *t* = 2.50, *p* = .013). Decision environment interacted with the three qualities (*b*_bias-free vs. agentic_ = 1.45, *t* = 10.11, *p* < .001; *b*_error-free vs. agentic_ = 1.07, *t* = 7.44, *p* < .001; *b*_bias-free vs. error-free_ = 0.38, *t* = 2.68, *p* = .008; Fig 7). Simple slope analysis showed that participants perceived that in the high-autonomy (vs. low-autonomy) environment agents were significantly more likely to display the agentic quality (*b* = -0.65, *t* = 5.77, *p* < .001), and were significantly less likely to display the bias-free (*b* = 0.80, *t* = 7.09, *p* < .001) and error-free (*b* = 0.42, *t* = 3.69, *p* < .001) qualities (Fig 7).

#### Perceived plausibility of the qualities

We conducted a mixed effects linear regression analysis on participants’ plausibility ratings for the qualities. Quality (agentic, bias-free, error-free) was included as a predictor. Random intercepts were included for participants to account for repeated measurements. The bias-free quality was rated as most plausible (*M* = 3.92), followed by the error-free (*M* = 3.52), and agentic (*M* = 2.79) qualities. There were significant differences between the bias-free and error-free qualities (*b* = -0.39, *t* = 5.03, *p* < .001) and between the error-free and agentic qualities (*b* = -0.73, *t* = 9.32, *p* < .001).

### Discussion

In the judgment task, participants weighted the error-free quality as more important than the agentic or bias-free qualities. Further replicating our findings Study 3, a preference for decisions by HSMs over decisions by ACPs was reversed by better ACP performance relative to HSMs on either the agentic, bias-free, or error-free qualities. Contrary to our hypotheses, participants were not influenced by the high vs. low autonomy of the decision environment in their weighting of the qualities. However, participants did report that agents were more likely to display the agentic quality in the high (vs. low) autonomy environment and were instead more likely to display the bias-free and error-free qualities in the low (vs. high) autonomy environment.

## General discussion

Who should decide how limited resources are prioritized? To answer this question, we investigated people’s perceptions of triaging decisions by autonomous computer programs (ACPs) and human staff members (HSMs) in a healthcare context. Studies 1a and 1b identified *agency*, *emotional experience*, *bias-free*, and *error-free* as four main qualities that describe people’s perceptions. Some of the qualities identified in the current investigation echo qualities of algorithms and humans discussed in the extant literature (e.g., *agency*, *emotional experience*, Gray et al., 2007; 2012, *bias*, [20, 21]). In the current investigation, these qualities emerged spontaneously from participants’ written accounts, providing a new source of evidence for their relevance to people’s perceptions. To our knowledge, no previous study has simultaneously compared participants’ perceptions of the multiple qualities for which algorithms and humans are perceived to differ. Indeed, HSMs and ACPs were not perceived to be equally capable of each quality. HSMs were perceived as more capable of displaying the agentic and emotional experience qualities, whereas ACPs were perceived as more capable of bias-free and error-free decision-making. In Studies 2–4, we used judgment tasks to measure the importance of the qualities in participants’ judgments of decisions by hypothetical HSMs and ACPs. These studies revealed that participants placed differing degrees of importance on the qualities. An important novel finding of our studies is that while participants showed a general preference for decisions by HSMs over decisions by ACPs, above average (Study 2) or relatively better (Studies 3 & 4) ACP performance, especially on qualities most characteristic of ACPs (i.e., error-free), was sufficient to reverse participants’ preferences in favor of decisions by ACPs. Previous studies have shown a preference for humans over algorithms even when participants are told that the overall performance of an algorithm is either equal to or *better* than the performance of a human decision maker [12]. Our studies show that when algorithms are compared to humans according to their various qualities, circumstances arise that favor algorithms over humans.

Bigman and Gray [11] found that participants were averse to decisions by ACPs compared to decisions by humans on the grounds that unlike humans, ACPs lack two important components of mind: agency (capacity to think, reason, & plan behavior; [20, 21]) and emotional experience (experience emotions, feelings, & empathy; [21]). In Study 1a, participants described their views of the qualities of ACPs and HSMs in response to open-ended questions. Our qualitative analysis of their written accounts identified agency and emotional experience as two of the main qualities underpinning people’s perceptions of decisions by ACPs and HSMs. This is an important finding as it shows that without guidance (i.e., using closed questioning) people’s attention is nonetheless drawn to an agent’s possession of characteristics of mind when reflecting on the agent’s capacity to make decisions that affect others (e.g., prioritizing patients according to their need). As such, agentic and emotional experience characteristics of mind appear to be perceived as inherent to an agent’s capacity to make appropriate moral decisions. We also found that HSMs were judged as more capable than ACPs of displaying agency and emotional experience (Study 1b), and that these qualities were more plausible in healthcare scenarios involving HSMs than scenarios involving ACPs (Study 2).

Our studies also identified bias-free and error-free decision-making among the main qualities underpinning people’s perceptions of ACPs and HSMs. Participants judged ACPs as more capable than HSMs of displaying bias-free and error-free qualities (Study 1b), and judged these qualities as more plausible in healthcare scenarios involving ACPs than scenarios involving HSMs. These findings have theoretical value for our understanding of people’s perceptions of autonomous technology. First, they suggest that people’s perceptions of the capabilities of ACPs (and HSMs) are complex and multifaceted, involving a diverse array of qualities that more often than not they do not consider in isolation. Second, they indicate that autonomous technology is perceived as possessing qualities (e.g., bias-free, error-free) that are less present in humans.

ACPs were perceived as capable of bias-free decision-making (Fig 1). This finding resonates with a tendency for algorithms to be perceived as processing information in a more decontextualized and de-differentiated manner [41], and for algorithms to be perceived as less motivated than humans toward prejudice [42]. Yet, while they may not possess the same tendencies toward prejudice and discrimination as humans [36], ACPs can exhibit similar biases due to the way the ACP is designed and characteristics of the data on which it is trained [38, 40]. In our studies, ACPs received high ratings for their capacity to make bias-free decisions. Stai and colleagues [56] found that fewer than half participants’ were comfortable with the idea of automated robotic surgery. Intriguingly, however, the majority of participants mistakenly believed that such autonomous technology is already used in surgery [56]. In general, people lack insight into how medical decisions are made by algorithms, perceiving them as a ‘black box’ [57]. People perceive algorithms as accurate information processors and as less capable of discrimination than humans, and even look to algorithms in situations where they anticipate discrimination [58]. Thus, public perceptions of technology may not align with the true capabilities and uses of artificial intelligence and automated technology. In connection to our current findings, in a healthcare context, as well as in other contexts, people may not be aware of potential sources of bias in autonomous technology.

ACPs have impressive computational power [4, 8], and like other computer-based systems, typically are also reliable and not prone to clerical errors [32]. Participants’ perceptions reflected these capabilities as they rated ACPs as both capable of error-free decision-making and more capable than HSMs (Fig 1). Intriguingly, however, participants identified the error-free quality also as a potential barrier to ACP acceptance. Our inspection of their written responses indicated that one concern among participants may have been that systematic error (e.g., in the computer code) could go undetected. Consequently, people who have initial reservations about ACPs may require reassurance about its track record or that it can learn to correct its errors. For instance, Berger et al. [59] found that participants were more receptive of advice from a fallible machine-learning-based IT support system when it demonstrated an ability to learn. In general, human error is typically perceived as random and correctable whereas erroneous decisions derived from statistical algorithms are typically perceived as systematic [60]. People are also less confident in algorithms that err than in humans that err, even when an algorithm outperforms a human [60, 61]. Thus, while people may perceive ACPs as less error prone than humans, they are also likely to judge an ACP more harshly than a human on its errors.

Our findings indicate that ACPs are perceived as potentially *better* decision makers than HSMs in their bias- and error-free capacities. Across Studies 2–4, participants prioritized error-free decision-making over other qualities in their judgments of hypothetical healthcare scenarios. In Study 2, emotional experience was treated as the least important quality. Therefore, while ACPs were not perceived to have a capacity for emotional experience, which resonates with the findings of other studies [11], other decision-making qualities were of greater priority to participants in their judgments of triaging decisions in our healthcare scenarios. In fact, participants’ general preference for decisions by HSMs over ACPs was reversed by above average (Study 2; Fig 3) or relatively better (Studies 3 & 4; Fig 6) ACP performance on other qualities, suggesting that emotional experience is not valued as highly as other qualities when it comes to triaging decisions.

As well as yielding theoretical insights into people’s perceptions of autonomous technology, our findings have practical implications. They suggest that to overcome barriers to patient adoption of autonomous technology their capacity for bias-free, and especially error-free, decision-making should be emphasized. ACPs possess some, but not all [20, 22], capacities for agency, afforded by their ability to create decision rules and draw inferences from machine learning algorithms [8, 30]. In Studies 2–4, displays of ACP agency were sufficient to reverse participants’ preference in favor of ACPs over HSMs. Emphasising the computational power of ACPs and their ability to make efficient and error-free decisions, may be an effective means of overcoming public reservations about autonomous technology.

With advances in artificial intelligence, future technology will exhibit greater abilities to make autonomous decisions. To address this emerging issue, in Study 4, we manipulated the level of autonomy afforded by the decision environment. Some participants were told that agents were required to follow a strict set of predetermined rules (*low-autonomy environment*), whereas others were told that agents were required to determine their own rules (*high-autonomy environment*). This method enabled us to manipulate the importance of decision qualities. We expected participants might prioritize the error-free quality in the former environment, due to the emphasis on implementing existing rules, and prioritize the agency and bias-free qualities in the latter environment, with the need to create new rules. We also expected that participants might show greater preference for decisions by HSMs in the high- vs. low-autonomy environment due HSMs greater perceived agency. Participants were not sensitive to the decision environment in the importance they placed on the qualities. However, they did judge that agents were more likely to display the agentic quality in the high (vs. low) autonomy environment and instead were more likely to display the bias-free and error-free qualities in the low (vs. high) autonomy environment (Fig 7). Our findings suggest that as the level of autonomy afforded by the environment increases, such as from automated computer-based systems that follow pre-programmed decision rules to more autonomous systems that derive their own rules, people expect more displays of agency, but also more biased decision-making and more error-prone decisions. Our findings also tentatively suggest that increases in the level of autonomy afforded by the environment may not necessarily alarm the public or raise new barriers to acceptance of technology.

In Studies 2–4, participants were shown how hypothetical ACPs and HSMs performed in a healthcare setting. In Study 2, participants imagined that ACPs or HSMs had been assessed by an independent review panel and had been awarded one to five stars based on their performance. We chose star ratings for two reasons: (1) star ratings are easier to understand for the layperson than contextual performance indices, such as statistics (e.g., percentage of patient referral requests completed within a specific period of time); and (2) similar metrics are used by independent regulators of health and social care in the United Kingdom. For example, the Care Quality Commission uses four ratings to evaluate health and social care services, including outstanding, good, requires improvement, and inadequate [62]. Ratings produced by regulators, such as the Care Quality Commission, are published for public consumption to help inform healthcare uses about the quality of public services.

Our study has limitations. We focussed on perceptions of autonomous technology in triaging decisions within a healthcare context. People’s perceptions are likely differ according to the decision domain (e.g., driving, legal, military) and the nature of the technology customary in a domain. Yet, in previous studies, aversion to autonomous technology making moral decisions was evident across a variety of domains [11, 12], indicating that people may possess partially domain-general perceptions autonomous technology. Second, our participant sample consisted of members of the public, rather than patients who are currently or have recently accessed healthcare. Nonetheless, the general public are primary stakeholder in national health services, and thus, drivers of change [9], and potentially the adoption of new technologies. We did not control for whether participants had experience working in healthcare. A fruitful avenue for future research would be to compare public perceptions with the perceptions of healthcare professionals who are likely to have more pragmatic and nuanced perspectives on autonomous technology [63]. The Study 1a (82%) and Study 1b (69%) samples contained a high proportion of female participants, which may have affected our item selection in our development of the Healthcare Decision Quality (HDQ) scale. However, Study 1c confirmed the HDQ scale factor structure with a separate sample of participants that was balanced in gender (52% female). We constructed a scale to capture the main qualities that describe people’s perceptions of algorithms and humans. A contribution of this approach is that while previous studies have typically focussed on a single quality in isolation, our approach distinguished four separate qualities that each play a role in people’s perceptions. Future research using different methods of recruitment and sample characteristics is required to establish further the psychometric properties of the scale. In Studies 3–4, we used a multiple-cue judgment task to examine people’s preferences for ACPs vs. HSMs when the two agents are pitted against each other and their relative performance can be directly compared. A viable alternative approach would have been to use a conjoint analysis. With a conjoint analysis, the performance of HSMs would be displayed alongside the performance of ACPs on each of the four cues. Our judgment task enabled a simpler presentation of information by displaying ACP performance relative to HSM performance. We displayed ACP performance relative to performance of HSMs (Fig 5), rather than vice versa, as ACPs provide an alternative to existing systems that consist of a human workforce. Our current focus is the potential barriers to ACP acceptance where ACPs may be perceived as less appropriate than HSMs for making decisions about healthcare provision. Future research could assess whether the framing of relative performance of ACPs versus HSMs influences people’s perceptions, which may provide useful insight into how best to frame ACP performance to promote its acceptance.

In conclusion, people’s perceptions of autonomous technology in a healthcare context comprise a variety of qualities. These qualities are not perceived to be equal in the decisions of HSMs and ACPs as the former are endorsed with human qualities of agency and emotional experience, whereas the latter are perceived to be more capable of bias- and error-free decision-making. Consequently, better than average, or relatively better ACP performance, especially on qualities most characteristic of ACPs, can be sufficient to reverse preference in favor of ACPs over HSMs. Decision-making agents were judged as more likely to display agency in a high (vs. low) autonomy environment, but also to be more biased and error-prone.

## Supporting information

S1 Appendix (DOCX)
