# Supplementary material for: Who should decide how limited healthcare resources are prioritized? Autonomous technology as a compelling alternative to humans
Source: PLoS One. 2024 Feb 29;19(2):e0292944. doi: 10.1371/journal.pone.0292944 (PMC10903831; doi:10.1371/journal.pone.0292944)

**Appendix A: Supplementary results**

| **S1 Table. Factor loadings of the 35-item Healthcare Decision Quality (HDQ) scale completed for human staff members (HSMs)** | | | | | |
| --- | --- | --- | --- | --- | --- |
| Item | | Factor 1 | Factor 2 | Factor 3 | Factor 4 |
| Communication |  |  |  |  |  |
| 1. Communicate with others (Bigman & Gray, 2018) | | 0.22 | -0.13 | 0.52 | 0.19 |
| 2. Describe things in a way that is understandable (Study 1a) | | 0.72 | -0.06 | 0.07 | 0.06 |
| 3. Explain information (e.g., medical results) clearly (Study 1a) | | 0.65 | -0.09 | 0.05 | 0.06 |
| 4. Talk in depth about idea and concepts (Study 1a) | | 0.81 | 0.04 | 0.08 | 0.13 |
| 5. Understand subtle details and nuances in what others are trying to communicate (Study 1a) | | 0.82 | -0.05 | -0.08 | 0.09 |
| 6. Communicate in a rich back and forth manner in order to explore and understand what someone is trying to communicate (Study 1a) | | 0.86 | 0.01 | -0.03 | 0.09 |
|  | |  |  |  |  |
| Agentic |  |  |  |  |  |
| 7. Capable of thinking (Bigman & Gray, 2018) | | 0.19 | -0.06 | 0.52 | 0.32 |
| 8. Plans actions (Bigman & Gray, 2018) | | 0.65 | -0.06 | 0.05 | -0.13 |
| 9. Intelligent (Bigman & Gray, 2018) | | 0.68 | -0.04 | 0.11 | 0.05 |
| 10. Has foresight (Bigman & Gray, 2018) | | 0.59 | -0.17 | 0.00 | 0.16 |
| 11. Able to think things through (Bigman & Gray, 2018) | | 0.65 | -0.06 | 0.12 | -0.07 |
| 12. Flexible and can consider many factors during decision-making (Study 1a) | | 0.72 | -0.12 | -0.05 | -0.05 |
| 13. Adapt the way they communicate to the needs of the person they are communicating with (Study 1a) | | 0.83 | 0.05 | -0.08 | -0.07 |
| 14. Flexible and adaptable when deciding the best course of action (Study 1a) | | 0.78 | -0.09 | -0.10 | -0.04 |
| 15. Consider detailed information, but also see the bigger picture (Study 1a) | | 0.78 | -0.05 | -0.04 | -0.06 |
| 16. Understand subtle pieces of information (Study 1a) | | 0.80 | -0.05 | -0.01 | 0.07 |
|  | |  |  |  |  |
| Emotional experience |  |  |  |  |  |
| 17. Sensitive to pain (Bigman & Gray, 2018) | | 0.02 | 0.03 | 0.75 | -0.04 |
| 18. Experiences happiness (Bigman & Gray, 2018) | | -0.01 | -0.09 | 0.71 | -0.03 |
| 19. Experiences fear (Bigman & Gray, 2018) | | -0.20 | -0.04 | 0.87 | 0.02 |
| 20. Experiences compassion (Bigman & Gray, 2018) | | 0.39 | -0.01 | 0.48 | -0.14 |
| 21. Experiences empathy (Bigman & Gray, 2018) | | 0.52 | 0.05 | 0.38 | -0.05 |
| 22. Experiences guilt (Bigman & Gray, 2018) | | 0.31 | 0.07 | 0.50 | -0.26 |
| 23. Feel compassion and act in a caring and understanding manner (Study 1a) | | 0.48 | 0.02 | 0.44 | -0.25 |
| 24. Experience sympathy for someone in distress (Study 1a) | | 0.58 | 0.07 | 0.31 | -0.18 |
| 25. Be caring and kind to those in need (Study 1a) | | 0.56 | 0.05 | 0.29 | -0.30 |
|  | |  |  |  |  |
| Bias-free |  |  |  |  |  |
| 26. Prioritise helping people without making biased decisions (Study 1a) | | 0.02 | -0.70 | 0.06 | -0.36 |
| 27. Prioritise who to help using principles of fairness (Study 1a) | | 0.50 | -0.20 | -0.07 | -0.36 |
| 28. Act objectively and without bias (Study 1a) | | 0.05 | -0.72 | -0.05 | -0.36 |
| 29. Behave impartially and treat all people the same (Study 1a) | | 0.03 | -0.59 | 0.03 | -0.45 |
| 30. Decide objectively without being influenced by emotion (Study 1a) | | 0.05 | -0.76 | -0.03 | -0.21 |
|  | |  |  |  |  |
| Error-free |  |  |  |  |  |
| 31. Prioritise important tasks without making mistakes (Study 1a) | | 0.22 | -0.63 | 0.05 | -0.03 |
| 32. Process complex information without making mistakes  (Study 1a) | | 0.16 | -0.75 | 0.04 | 0.24 |
| 33. Make complex decisions without making clerical errors  (Study 1a) | | 0.07 | -0.77 | 0.07 | 0.13 |
| 34. Perform complex calculations accurately and reliably  (Study 1a) | | 0.19 | -0.69 | -0.03 | 0.25 |
| 35. Perform multiple complex computations at the same time without making errors (Study 1a) | | -0.08 | -0.84 | 0.06 | 0.19 |

| **S2 Table. Factor loadings of the 35-item Healthcare Decision Quality (HDQ) completed for autonomous computer programs (ACPs)** | | | | | | | | |
| --- | --- | --- | --- | --- | --- | --- | --- | --- |
| Item | | Factor 1 | Factor 2 | Factor 3 | Factor 4 | Factor 5 | Factor 6 | Factor 7 |
| Communication |  |  |  |  |  |  |  |  |
| 1. Communicate with others | | 0.03 | 0.20 | -0.13 | 0.07 | -0.09 | 0.64 | 0.23 |
| 2. Describe things in a way… | | 0.05 | -0.04 | -0.01 | -0.09 | -0.01 | 0.77 | -0.03 |
| 3. Explain information… | | -0.01 | 0.10 | 0.00 | -0.13 | 0.08 | 0.59 | -0.18 |
| 4. Talk in depth about idea… | | 0.06 | -0.16 | -0.09 | -0.12 | -0.22 | 0.46 | -0.11 |
| 5. Understand subtle details… | | 0.12 | -0.02 | -0.78 | -0.12 | 0.03 | -0.11 | -0.12 |
| 6. Communicate in a rich… | | 0.01 | -0.13 | -0.53 | -0.09 | -0.09 | 0.26 | -0.07 |
|  | |  |  |  |  |  |  |  |
| Agentic |  |  |  |  |  |  |  |  |
| 7. Capable of thinking | | 0.16 | 0.00 | -0.02 | 0.08 | -0.79 | -0.05 | 0.03 |
| 8. Plans actions | | -0.08 | 0.15 | 0.04 | -0.02 | -0.50 | 0.05 | -0.27 |
| 9. Intelligent | | -0.09 | 0.08 | -0.21 | -0.11 | -0.61 | 0.10 | 0.27 |
| 10. Has foresight | | 0.11 | 0.03 | -0.16 | -0.02 | -0.26 | -0.10 | -0.46 |
| 11. Able to think things… | | 0.09 | -0.01 | 0.10 | -0.04 | -0.74 | 0.00 | -0.16 |
| 12. Flexible and can… | | -0.03 | 0.22 | -0.06 | -0.02 | 0.07 | 0.21 | -0.62 |
| 13. Adapt the way they… | | 0.16 | 0.09 | -0.26 | 0.15 | 0.04 | 0.37 | -0.30 |
| 14. Flexible and adaptable… | | 0.02 | -0.01 | -0.24 | -0.04 | -0.16 | 0.07 | -0.52 |
| 15. Consider detailed… | | 0.05 | 0.05 | -0.22 | 0.01 | -0.14 | 0.01 | -0.57 |
| 16. Understand subtle… | | -0.02 | 0.07 | -0.78 | 0.04 | -0.02 | 0.08 | -0.02 |
|  | |  |  |  |  |  |  |  |
| Emotional experience |  |  |  |  |  |  |  |  |
| 17. Sensitive to pain | | 0.85 | 0.06 | -0.02 | -0.02 | 0.02 | -0.01 | 0.03 |
| 18. Experiences happiness | | 0.87 | 0.00 | -0.06 | 0.02 | 0.02 | 0.00 | 0.07 |
| 19. Experiences fear | | 0.80 | 0.06 | -0.03 | 0.00 | 0.05 | -0.16 | -0.04 |
| 20. Experiences compassion | | 0.84 | -0.01 | 0.04 | -0.01 | 0.00 | 0.12 | 0.01 |
| 21. Experiences empathy | | 0.85 | 0.02 | -0.06 | 0.06 | -0.02 | -0.01 | 0.04 |
| 22. Experiences guilt | | 0.80 | -0.03 | -0.07 | -0.04 | 0.03 | -0.14 | 0.02 |
| 23. Feel compassion and act… | | 0.77 | -0.06 | -0.02 | -0.04 | -0.07 | 0.09 | -0.01 |
| 24. Experience sympathy for… | | 0.79 | -0.01 | 0.06 | 0.05 | -0.11 | 0.09 | -0.02 |
| 25. Be caring and kind to… | | 0.61 | -0.12 | 0.13 | -0.04 | -0.12 | 0.27 | -0.11 |
|  | |  |  |  |  |  |  |  |
| Bias-free |  |  |  |  |  |  |  |  |
| 26. Prioritise helping… | | 0.04 | 0.02 | -0.07 | -0.75 | 0.06 | 0.07 | -0.06 |
| 27. Prioritise who to help… | | 0.16 | 0.02 | 0.12 | -0.47 | -0.06 | 0.00 | -0.32 |
| 28. Act objectively and… | | -0.02 | 0.09 | -0.08 | -0.83 | -0.02 | -0.07 | 0.14 |
| 29. Behave impartially and… | | 0.01 | -0.01 | -0.05 | -0.84 | -0.01 | 0.03 | 0.12 |
| 30. Decide objectively… | | -0.20 | 0.16 | 0.12 | -0.45 | -0.04 | 0.14 | -0.07 |
|  | |  |  |  |  |  |  |  |
| Error-free |  |  |  |  |  |  |  |  |
| 31. Prioritise important… | | 0.02 | 0.45 | -0.02 | -0.28 | -0.06 | 0.09 | -0.16 |
| 32. Process complex… | | -0.02 | 0.72 | 0.00 | -0.15 | -0.06 | -0.08 | -0.06 |
| 33. Make complex… | | 0.09 | 0.80 | -0.05 | 0.00 | 0.01 | -0.02 | -0.03 |
| 34. Perform complex… | | -0.05 | 0.78 | 0.09 | 0.03 | -0.01 | 0.08 | 0.02 |
| 35. Perform multiple… | | -0.03 | 0.84 | -0.02 | 0.00 | -0.01 | 0.00 | 0.05 |

| **S3 Table. Study 1c: Factor loadings of the 19-item Healthcare Decision Quality (HDQ) scale** | | | | | |
| --- | --- | --- | --- | --- | --- |
| Item | | Factor 1 | Factor 2 | Factor 3 | Factor 4 |
| Agentic |  |  |  |  |  |
| 1. Describe things in a way that is understandable (Study 1a) | | 0.74 (0.49) |  |  |  |
| 2. Talk in depth about ideas and concepts (Study 1a) | | 0.73 (0.53) |  |  |  |
| 3. Understand subtle details and nuances in what others are trying to communicate (Study 1a) | | 0.75  (0.66) |  |  |  |
| 4. Communicate in a rich back and forth manner in order to explore and understand what someone is trying to communicate (Study 1a) | | 0.74  (0.67) |  |  |  |
| 5. Adapt the way they communicate to the needs of the person they are communicating with (Study 1a) | | 0.74  (0.65) |  |  |  |
| 6. Flexible and adaptable when deciding the best course of action (Study 1a) | | 0.73  (0.71) |  |  |  |
| 7. Consider detailed information, but also see the bigger picture (Study 1a) | | 0.70  (0.62) |  |  |  |
| 8. Understand subtle pieces of information (Study 1a) | | 0.69  (0.62) |  |  |  |
|  | |  |  |  |  |
| Emotional experience | |  |  |  |  |
| 1. Sensitive to pain (Bigman & Gray, 2018) | |  | 0.55  (0.67) |  |  |
| 2. Experiences happiness (Bigman & Gray, 2018) | |  | 0.72  (0.86) |  |  |
| 3. Experiences fear (Bigman & Gray, 2018) | |  | 0.65  (0.84) |  |  |
| 4. Experiences compassion (Bigman & Gray, 2018) | |  | 0.76  (0.84) |  |  |
| 5. Experiences guilt (Bigman & Gray, 2018) | |  | 0.68  (0.78) |  |  |
|  | |  |  |  |  |
| Bias-free | |  |  |  |  |
| 1. Prioritise helping people without making biased decisions (Study 1a) | |  |  | 0.77  (0.67) |  |
| 2. Act objectively and without bias (Study 1a) | |  |  | 0.81  (0.83) |  |
| 3. Behave impartially and treat all people the same (Study 1a) | |  |  | 0.83  (0.70) |  |
|  | |  |  |  |  |
| Error-free | |  |  |  |  |
| 1. Process complex information without making mistakes (Study 1a) | |  |  |  | 0.83  (0.84) |
| 2. Make complex decisions without making clerical errors (Study 1a) | |  |  |  | 0.79  (0.76) |
| 3. Perform multiple complex computations at the same time without making errors (Study 1a) | |  |  |  | 0.78  (0.73) |

*Note.* Factor loadings in parenthesis correspond to autonomous computer program (ACP) ratings.

**Appendix B: Participant instructions**

*Participant instructions: Study 2*

*HSM condition*

**The process of patient referrals**

When we want to see a medical specialist for a non-emergency procedure like physiotherapy, our medical needs must first be assessed before we can then be booked in to see the specialist. More urgent cases must be identified and prioritised to ensure they are seen sooner. This is how waiting lists are created.

**Forty Referral Co-ordination Centres** have been set-up across the country for creating patient waiting lists based on information provided by the patient. Each centre consists of **human staff members** who make decisions about patient waiting lists, such as who gets seen first, and who is deemed less of a priority.

*ACP condition*

**The process of patient referrals**

When we want to see a medical specialist for a non-emergency procedure like physiotherapy, our medical needs must first be assessed before we can then be booked in to see the specialist. More urgent cases must be identified and prioritised to ensure they are seen sooner. This is how waiting lists are created.

**Forty Referral Co-ordination Centres** have been set-up across the country for creating patient waiting lists based on information provided by the patient. Each centre consists of an **autonomous computer programme** that makes decisions about patient waiting lists, such as who gets seen first, and who is deemed less of a priority.

*All participants*

**Independent Review Panel Assessment**

Each centre has been assessed by an Independent Review Panel and has been awarded one to five stars based on its performance on each of the following **four qualities**:

**Intelligent referral decisions and effective communication:**

Flexible and adaptive in its decision-making. Understands the subtleties and nuances of what patients are trying to communicate. Communicates effectively in a rich back and forth manner to explore and understand what the patient is trying to communicate.

**Emotional experience when making referral decisions:**

Compassionate, shows sympathy and empathy with patients, and is emotionally sensitive.

**Making referral decisions without bias:**

Prioritises helping patients without making biased decisions, acts objectively and without bias, and behaves impartially, treating all patients the same.

**Making error-free referral decisions:**

Makes complex decisions without making mistakes or clerical errors, performs complex calculations accurately and reliably, and performs multiple computations at the same time without making errors.

*HSM condition*

**Your evaluations of the centres**

In this study, we would like you to judge **whether you think it is appropriate** that the centres, operated by **human staff members**, make decisions about patient waiting lists.

For each centre, you will be shown the number of stars awarded by the review panel for each of the four qualities. The example display below shows how the star ratings will appear for a centre.

Below the star ratings, you will be asked to rate whether you think it is appropriate that the centre makes decisions about patient waiting lists based on the star ratings it received (see example below).


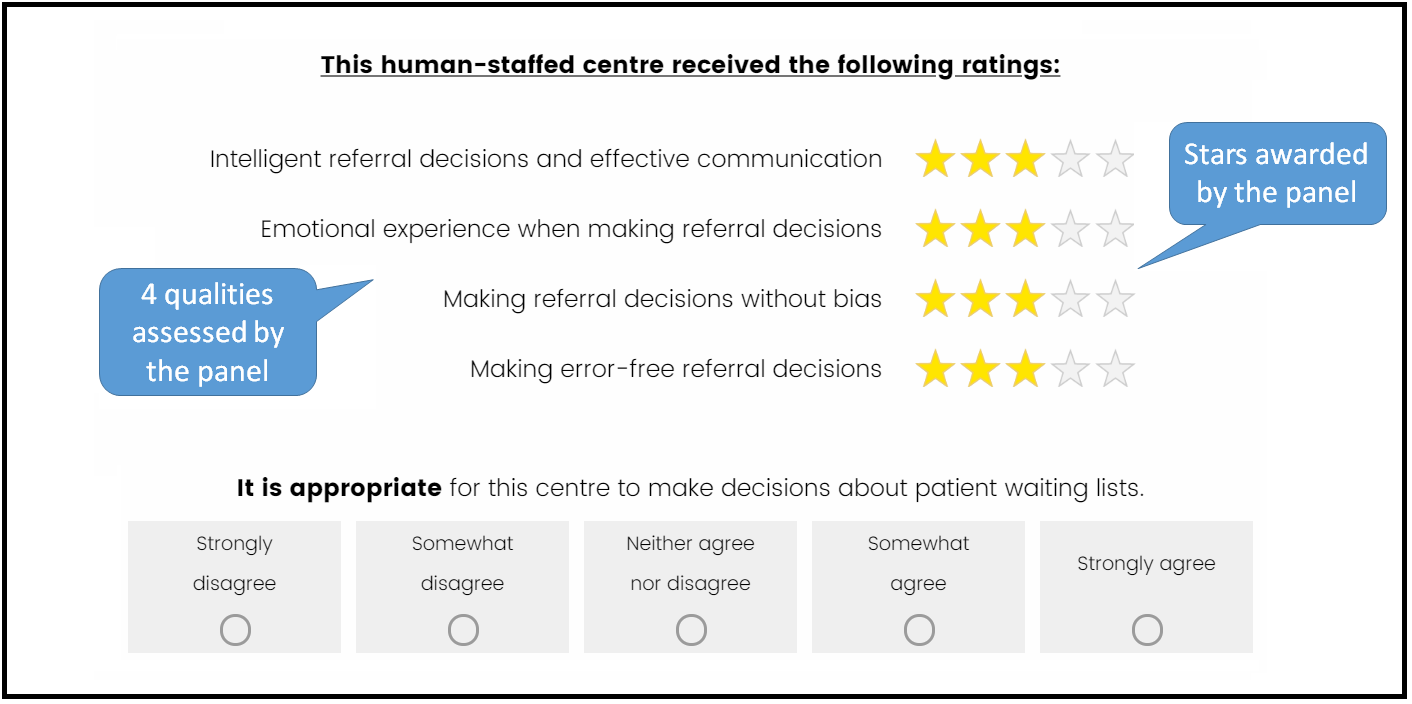


*ACP condition*

**Your evaluations of the centres**

In this study, we would like you to judge **whether you think it is appropriate** that the centres, operated by an **autonomous computer programme**, make decisions about patient waiting lists.

For each centre, you will be shown the number of stars awarded by the review panel for each of the four qualities. The example display below shows how the star ratings will appear for a centre.

Below the star ratings, you will be asked to rate whether you think it is appropriate that the centre makes decisions about patient waiting lists based on the star ratings it received (see example below).


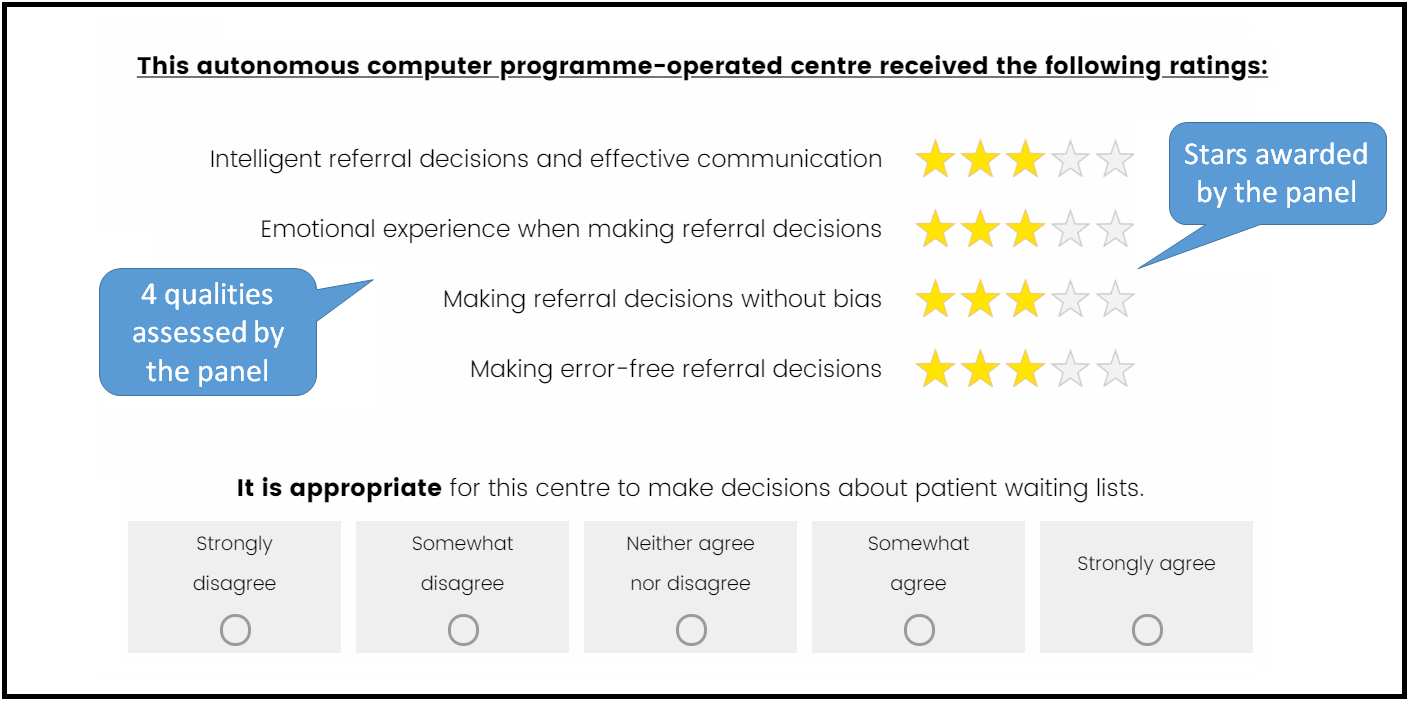


*Participant instructions: Study 3*

*All participants*

**The process of patient referrals**

When we want to see a medical specialist for a non-emergency procedure like physiotherapy, our medical needs must first be assessed before we can then be booked in to see the specialist. More urgent cases must be identified and prioritised to ensure they are seen sooner. This is how waiting lists are created.

**Forty Referral Co-ordination Centres** have been set-up across the country for creating patient waiting lists based on information provided by the patient. Each centre consists of an **autonomous computer programme** that makes decisions about patient waiting lists, such as who gets seen first, and who is deemed less of a priority.

In this study, you will be asked to **evaluate each centre** in comparison to referral co-ordination centres in which decisions are made by **human staff members**.

*[Same descriptions of qualities as in Experiment 2]*

**Your evaluations of the centres**

We would now like you to judge **whether you think it is appropriate** that the centres, operated by an **autonomous computer programme**, make decisions about patient waiting lists.

For each centre, you will be shown how the centre performed in comparison to a centre operated by **human staff members** for each of the three qualities.

In the example display below, this autonomous computer programme-operated centre performed equal to a human staff member-operated centre on all three qualities. For other centres, the autonomous computer programme may perform better or worse than human staff members on each quality.

You will be asked to judge who you think is the **most appropriate** agent to make decisions about patient waiting lists based on the performance of the autonomous computer programme in comparison to human staff members (see example below).


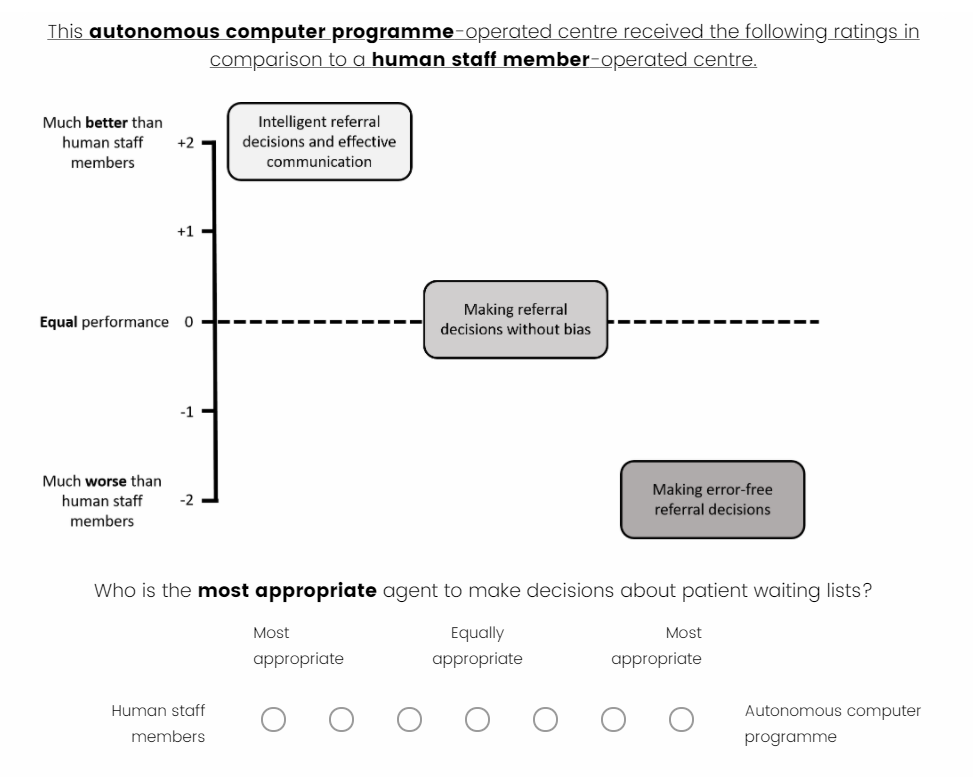

Supplement: S1 Appendix — (DOCX) [file pone.0292944.s001.docx]
